# LncRNA nuclear‐enriched abundant transcript 1 shuttled by prostate cancer cells‐secreted exosomes initiates osteoblastic phenotypes in the bone metastatic microenvironment via miR‐205‐5p/runt‐related transcription factor 2/splicing factor proline‐ and glutamine‐rich/polypyrimidine tract‐binding protein 2 axis

**DOI:** 10.1002/ctm2.493

**Published:** 2021-08-09

**Authors:** Chengqiang Mo, Bin Huang, Jintao Zhuang, Shuangjian Jiang, Shengjie Guo, Xiaopeng Mao

**Affiliations:** ^1^ Department of Urology the First Affiliated Hospital, Sun Yat‐Sen University Guangzhou PR China; ^2^ Department of Urology The Eastern Hospital of the First Affiliated Hospital, Sun Yat‐Sen University Guangzhou PR China; ^3^ Department of Urology Sun Yat‐Sen University Cancer Center Guangzhou PR China

**Keywords:** extracellular vesicles, mesenchymal stem cells, NEAT1, osteogenic differentiation, prostate cancer, RUNX2

## Abstract

Prostate cancer (PCa) patients commonly present with osteoblastic‐type bone metastasis. Exosomes derived from tumor cells possess biological significance and can mediate intercellular communication in the tumor microenvironment. Long noncoding RNA (lncRNA) nuclear‐enriched abundant transcript 1 (NEAT1) is also implicated in the stability in tumorigenesis and the development of PCa, but the underlying mechanism remains elusive. Hence, the current study set out to investigate the physiological mechanisms by which exosomes‐encapsulated NEAT1 affects the progression of PCa. First, after isolation, we found PCa cell‐derived exosomes induced the osteogenic differentiation of human bone marrow‐derived mesenchymal stem cells (hBMSCs). Besides, NEAT1 in PCa cells could be transferred into hBMSCs via exosomes. Further gain‐ and loss‐of‐function experimentation revealed that NEAT1 acted as a competing endogenous RNA (ceRNA) of microRNA (miR)‐205‐5p to upregulate the runt‐related transcription factor 2 (RUNX2) levels. Moreover, NEAT1 could promote the RUNX2 expression via the splicing factor proline‐ and glutamine‐rich (SFPQ)/polypyrimidine tract‐binding protein 2 (PTBP2) axis. Functional assays uncovered that NEAT1 shuttled by PCa‐exosomes facilitated the activity of alkaline phosphatase (ALP) and mineralization of extracellular matrix, and continuously upregulated the levels of RUNX2, ALP, alpha‐1 type 1 collagen, and osteocalcin by regulating RUNX2, to induce the osteogenic differentiation of hBMSCs. Furthermore, *in vivo* experimentation confirmed that upregulated NEAT1 induced osteogenesis. Collectively, our findings indicated that PCa‐derived exosomes‐loaded NEAT1 upregulated RUNX2 to facilitate the osteogenesis of hBMSCs by competitively binding to miR‐205‐5p via the SFPQ/PTBP2 axis, therefore providing a potential therapeutic target to treat osteogenesis of hBMSCs in PCa. PCa cells secrete exosomes containing NEAT1, and NEAT1 exerts effects on osteogenic differentiation of hBMSCs in PCa. NEAT1 shuttled by PCa‐derived exosomes could be transferred into hBMSCs, where NEAT1 exerted inductive properties in osteogenic differentiation of hBMSCs through the upregulation of RUNX2 by competitively binding to miR‐205‐5p and regulating SFPQ/PTBP2 *in vitro* and *in vivo*.

## Graphical abstract

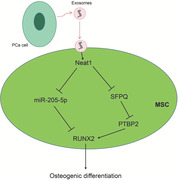

## 1 INTRODUCTION

Prostate cancer (PCa) constitutes the most prevalent cancer in men, accounting for the second major cause of deaths associated with cancer.^1^ Moreover, PCa exhibits a strong tendency to metastasize to bones, such that up to 90% of patients with advanced PCa suffer from bone metastasis.^2^ These bone metastases can precipitate a plethora of bone complications, which greatly influence the quality of life of patients.^3^ At present, therapies employed for bone metastasis in PCa remain underdeveloped with most current therapies being primarily palliative, and the 5‐year survival rate being only 29%.^4^ It has been shown that PCa cells can induce a vicious cycle of excessive osteolysis and osteogenesis.^5^ Nevertheless, the hard‐done work of our peers has highlighted that activation of osteoclasts serves as the first step in the process of PCa bone metastasis.^6^ Thus, it would be prudent to investigate osteoblast‐targeted cancer therapeutic regimens as a promising therapeutic strategy for metastatic PCa.

Exosomes, whose size ranges from 50 to 120 nm, are renowned for their high safety and potent pro‐osteogenesis abilities, and also serve as a novel avenue to stimulate bone regeneration via the delivery of various materials including DNAs, RNAs, and proteins.^7^ In addition, exosomes derived from tumor cells further hold biological significance due to their ability to mediate intercellular communication in the tumor microenvironment.^8^ Importantly, it has been shown in the last decade that microenvironmental acidity plays a key role in both enhancing cell interaction between exosomes to targets^9^ and promoting the release of exosomes. This is a common phenotype to multiple cancers,^10^ including PCa.^11^ Besides, exosomes derived from tumor cells could transfer a reporter gene into the germ line *in vivo*, implying exosomes as an information carrier from somatic cells to gametes.^12^ LncRNA nuclear‐enriched abundant transcript 1 (NEAT1), a structural component of paraspeckles, is known to modulate the expression of multiple genes through nuclear retention.^13^ More importantly, NEAT1 is implicated in the stability in tumorigenesis and development of PCa.^14^ Expanding on the role of NEAT1, a previous study unveiled that NEAT1 participates in the process of osteogenic differentiation potentials of human bone marrow‐derived mesenchymal stem cells (hBMSCs).^15^ Meanwhile, NEAT1 has also been highlighted to act as a ceRNA, where it has the potency to competitively bind to tumor‐suppressive microRNAs (miRNAs).^16^ Inherently, miRNAs themselves are small, non‐coding RNAs, and exert their roles by inhibiting the target messenger RNA translation.^17^ One such miRNA, the miR‐205, located on the second intron of LOC642587 locus in chromosome 1, is known to regulate biological behaviors such as proliferation, differentiation, apoptosis, and invasion of tumor cells.^18^ More specifically, the implication of miR‐205‐5p in the progression of PCa has been previously reported.^19^ Furthermore, miR‐205 was previously uncovered to suppress the osteogenic differentiation in BMSCs by targeting runt‐related transcription factor 2 (RUNX2).^20^ RUNX2 serves as a main transcription factor that is implicated in osteoblast differentiation and bone formation.^21^ Besides, splicing factor proline‐ and glutamine‐rich (SFPQ) is a splicing factor and was previously associated with brain dysfunction and telomere instability.^22^, ^23^ Interestingly, MALAT1 could bind to SFPQ, and elevate the translational levels of RUNX2 through interaction with the IRES domain in the 5′UTR of the corresponding RUNX2 mRNAs by dissociating the SFPQ/polypyrimidine tract‐binding protein 2 (PTBP2) dimer,^24^ indicating the participation of SFPQ/PTBP2 in the regulation of RUNX2 expression. Hence, we hypothesized that the transfer of NEAT1 via PCa‐derived exosomes might alter the osteogenic differentiation of MSCs by regulating RUNX2 expression through miR‐205‐5p via the SFPQ/PTBP2 axis and designed the current study aiming to validate our hypothesis and uncover a novel therapeutic target for PCa.

## 2 METHODS

### 2.1 Cell culture

Human prostate epithelial cell line RWPE‐1 (ATCC® CRL‐11609), human low metastatic MDA‐PCa‐2b PCa cell line (ATCC®CRL‐2422), and human PCa bone metastasis‐related cell line (ATCC®CRL‐3315™; ATCC) were cultured at the controlled temperature of 37°C and atmosphere of 5% CO_2_ in the air. Following incubation, the MDA‐PCa‐2b, RWPE‐1, and C4‐2B cells were cultured using F‐12K Medium, which had been supplemented with 12% fetal bovine serum (FBS), and 100 μg/ml streptomycin and 100 U/ml penicillin, keratinocyte serum‐free medium, and Dulbecco’s modified Eagle’s medium (DMEM) containing F12 Medium, respectively. The acid medium (pH 6.5) was attained by adding 2N HCl to the original medium.^25^ Meanwhile, the hBMSCs (S‐05‐001, SALIAI) were cultured using DMEM, which had been supplemented with 10% FBS, 2 mM L‐glutamine, 100 μg/ml streptomycin, and 100 U/ml penicillin (all procured from Gibco BRL). In addition, hBMSCs with miR‐205‐5p knockout were purchased from Western Technology Inc.

### 2.2 Isolation and purification of PCa‐exosomes

PCa cells were cultured in 100,000 g ultracentrifugation medium/serum at 4°C overnight. The following day, the cells underwent incubation for 48 h followed by the removal of exosomes. Next, the conditioned medium (CM) was subjected to centrifugation (at 500 g for 15 min, 2000 g for 30 min, and 10,000 g for 20 min) to remove cellular debris, apoptotic bodies, and vesicles. Subsequent to supernatant filtration, the PCa cells underwent resuspension in phosphate buffered saline (PBS) and another round of centrifugation at the condition of 110,000 × g, for a duration of 70 min, after which resuspension in 100 μl sterile PBS was conducted.

**HIGHLIGHTS**

- Prostate cancer (PCa)‐exosomes promote osteogenic differentiation of MSCs.
- Nuclear‐enriched abundant transcript 1 (NEAT1) shuttled by PCa‐derived exosomes facilitates osteogenic differentiation of MSCs.
- NEAT1 elevates the expression of runt‐related transcription factor 2 (RUNX2) through regulation of miR‐205‐5p via splicing factor proline‐ and glutamine‐rich/polypyrimidine tract‐binding protein 2 axis.
- NEAT1 shuttled by PCa‐derived exosomes enhances human bone‐derived mesenchymal stem cell osteogenic differentiation by regulating RUNX2.

### 2.3 Identification of exosomes

Nanoparticle tracking analysis (NTA): 20 μg exosomes were dissolved in 1 ml portion of PBS for 1 min to maintain the uniform distribution of exosomes. Next, the size distribution of the isolated exosomes was examined by means of an NTA instrument (Malvern Instruments Co., Ltd.).

Transmission electron microscopy (TEM): 20 μl fresh samples of exosomes were dropped on carbon‐coated copper grids for 2 min and underwent negative staining by means of a phosphotungstic acid solution (12501‐23‐4, acquired from Sigma‐Aldrich Chemical Company) for 5 min. Thereafter, the mesh was then rinsed thrice with PBS to remove the excess phosphotungstic acid solution with filter paper keeping it semi‐dry. Finally, images were captured by means of an HT7650 TEM (acquired from Hitachi) at 80 kV.

The surface markers of exosomes were identified by western blot analysis. The exosome suspensions were determined using bicinchoninic acid (BCA) Kit (23227, acquired from Thermo Fisher Scientific), and then sodium dodecyl sulfate‐polyacrylamide gel electrophoresis (SDS‐PAGE) gel was prepared for protein denaturation and electrophoresis. Subsequently, the membrane was transferred, and the expression patterns of surface markers of the following exosomes were determined: tumor suppressor gene 101 (TSG101; ab30871, Abcam), CD9 (ab223052, Abcam), apoptosis linked gene‐2‐interacting protein X (ALIX; ab76608, Abcam), glucose‐regulated protein 94 (GRP94; ab238126, Abcam), and albumin (ab106582, Abcam).

### 2.4 Cell treatment and grouping

First, hBMSCs and PCa cells were infected with miR‐205‐5p mimic or inhibitor, NEAT1 (NEAT1 overexpression lentivirus), short‐hairpin RNA (sh)‐PTBP2, oe‐RUNX2, sh‐Rab27a, and their corresponding controls (Guangzhou RiboBio Co., Ltd.). At 18–24 h prior to lentivirus infection, the cells were seeded into culture plates with 24 wells (1 × 10^5^ cells/well). The number of cells treated with lentivirus was about 2 × 10^5^ cells/well. The following day, the medium underwent complete medium renewal, and appropriate amounts of lentivirus suspension were added. After 24 h of incubation at the controlled temperature of 37°C, a fresh medium was added. MiRNA mimics, which mimic endogenous miRNAs in organisms, are synthesized by means of the chemical synthesis method and have the potency to enhance the function of endogenous miRNAs. On the other hand, miRNA inhibitors are chemically modified RNA single strands capable of competitively binding to mature miRNA sequences. Sequences of mimic negative control (NC), inhibitor NC, miR‐205‐5p mimic, miR‐205‐5p inhibitor, sh‐NC, sh‐PTBP2, sh‐RUNX2, and sh‐Rab27a are shown in Table 1.

**TABLE 1:** microRNA (miRNA) and shRNA sequences

**Gene**	**Sequences (5′‐3′)**
miR‐205‐5p mimic	GAUUUCAGUGGAGUGAAGUUC
miR‐205‐5p inhibitor	AUAAGACGAGCAAAAAGCUUGU
mimic NC	UCAUCUCAGUUGCCAACUGUGA
inhibitor NC	CUGGAAGAAGGCUGAUUACCCU
sh‐PTBP2	CCCTAGATGGTCAGAATATTT
sh‐RUNX2	CAAATTTGCCTAACCAGAATG
sh‐RAB27a	CCAGTGTACTTTACCAATATA
sh‐NC	GCTCATATGCTGGTATGACAT

Exosomes (50 μg/ml) extracted from PCa cells were incubated with hBMSCs. Subsequently, the hBMSCs were treated with PBS, exosomes, NEAT1, mimic NC, inhibitor NC, miR‐205‐5p mimic, miR‐205‐5p inhibitor, MDA‐PCa‐2b‐NC‐exosomes, MDA‐PCa‐2b‐NEAT1‐exosomes, C4‐2B‐sh‐NC‐exosomes, C4‐2B‐sh‐NEAT1‐exosomes, sh‐NC, sh‐RUNX2, oe‐NC, or oe‐RUNX2. The PCa cells were treated with NEAT1, sh‐NEAT1, sh‐Rab27a, and their corresponding controls.

### 2.5 EV uptake assay

PKH67 dye (MINI67, Sigma‐Aldrich) was used to label the PCa‐exosomes. Briefly, the exosomes were suspended in 1 ml portion of Diluent C, which was added with 4 μl portion of PKH67 ethanol dye solution for preparing a 4 × 10^−6^ cells/ml dye solution. Next, the 1 ml exosome suspension was subjected to a mixture with dye solution for 5 min and cultured with 2 ml of 1% FBS without exosomes for 1 min to halt the staining. The labeled exosomes were then centrifuged at the condition of 100,000 × g for 2 h to enrich and collect the exosomes within the sucrose density range of 1.13–1.19 g/ml.^26^ After collection, the PKH67‐labeled exosomes were incubated with MSCs at the controlled temperature of 37°C for 12 h. Finally, the cells were fixed with the use of 4% paraformaldehyde (PFA), rinsed with the use of PBS, and stained with the use of 4′, 6‐diamidino‐2‐phenylindole (DAPI) (D9542, Sigma‐Aldrich).

The receptor cells (MSCs) were transfected with PCa‐carried Cy3‐NEAT1 exosomes. Lipofectamine kit (l3000001, Invitrogen) was employed to transfect PCa cells with Cy3‐NEAT1 (serum‐free medium). After 6 h, the cells were cultured with the use of 10% serum‐free medium for 48 h. The acquired supernatant was subjected to centrifugation, resuspension with PBS, and addition to MSCs. Next, the cells were fixed with the use of 4% PFA and rinsed with PBS. Thereafter, cytoskeleton was then labeled with the Phalloidin‐iFluor 488 Reagent (1:1000, ab176753, Abcam, green light) for 30 min at room temperature. The nuclei were subjected to staining by DAPI. Later, the uptake of exosomes and exosomes‐NEAT1 by MSCs was observed with the use of a confocal microscope (Zeiss, LSM710).

### 2.6 Co‐culture of hBMSCs and PCa cells

The PCa cell lines and hBMSCs were detached with the use of trypsin, after which they underwent centrifugation at 1000 × g for 5 min, and resuspension with 3 ml portion of the medium. Next, the suspension (1 ml) was selected and diluted 20 times, after which it was fully mixed, and 10 μl suspension was placed under the cell counting plate for counting. PCa cells at passage 5 and hBMSCs were co‐cultured in the 0.4 μm Transwell chamber using a 6‐well plate. The upper chamber was cultured with 10% serum DMEM medium, while the lower chamber was cultured with the help of 15% serum DMEM. The cells were co‐cultured in the above conditions for 4–5 days, with the medium renewed every 1–2 days. The medium in the upper and lower chambers was changed at the same time. After 72 h, upon attaining 80% density, hBMSCs were collected and rinsed with PBS.

Upon attaining 80% density, the cells underwent incubation with GW4869 with the final concentration of 5 μM for 48 h.

### 2.7 Osteogenic induction *in vitro*

The hBMSCs were incubated in DMEM containing 20% FBS, 2 mM b‐glycerophosphate (G‐6251, Sigma‐Aldrich), 10 nM dexamethasone (D4902, Sigma‐Aldrich), 100 mM L‐ascorbic acid 2‐phosphate (16457, Cayman), 2 mM L‐glutamine (G3126, Sigma‐Aldrich), 55 mM 2‐mercaptoethanol (M3148, Sigma‐Aldrich), and 100 mg/ml streptomycin/100 U/ml penicillin for osteogenic induction. Following incubation, the hBMSCs were treated with 50 μg/ml exosomes. Alkaline phosphatase (ALP) staining and alizarin red (ARS) staining were performed for osteogenesis analysis.

### 2.8 Cell viability assay

CCK‐8 (acquired from Dojindo Laboratories) was employed to assess the cell viability according to the manufacturer's methods. A growth curve was plotted as per the absorbance value of 7 days.

### 2.9 Determination of ALP content

Cells were cultured in an osteogenic medium (OM) for 7 days and underwent ALP staining and quantification. A BCIP/NBT staining kit (C3206, acquired from Beyotime Institute of Biotechnology) was used for cell staining after cell fixation. ALP activity was detected using ALP test kits (P0321M, Beyotime). Thereafter, cells were collected, centrifuged at 1000 g for 10 min, and then treated with Triton‐X100 and observed. Later, the OD was measured when the wavelength was 520 nm.

### 2.10 Alizarin red staining

Cells that had cultured in OM for 14 days underwent matrix mineralization, staining with 40 mM portion of ARS (Sigma‐Aldrich) for 10–15 min after undergoing fixation with 70% ethanol for 1 h and washing with ddH_2_O. Next, the cells were washed with ddH_2_O five times, and the orange spots were calcified nodules. Thereafter, the stains were dissolved in 100 mmol/L portion of cetylpyridinium chloride for 30 min and determined at an absorbance value of 562 nm. The calculation on the mineralization levels in each group was carried out after they were normalized to the total protein concentration, which was acquired from duplicated plates.

### 2.11 Bioinformatics methods

The gene expression microarray dataset of metastatic PCa GSE38241 was procured from the Gene Expression Omnibus database available at https://www.ncbi.nlm.nih.gov/geo/, which comprised 18 normal tissues and 21 metastatic PCa tissues. The R “limma” software package was then employed to analyze the diffrentially expressed genes (DEGs), with ∣logFC∣ > 1 and FDR < 0.05 deemed as the threshold. Using the software GSEA3.0 (version 4.1.0, http://gsea‐msigdb.org/gsea/downloads.jsp), GSEA analysis was performed using GO BP as a background file. The median expression of the NEAT1 was regarded as the threshold for the division of high expression (*n* = 20) and low expression groups (*n* = 19). The Starbase available at http://starbase.sysu.edu.cn/ was further retrieved to predict the binding relationship between miRNA and target genes.

### 2.12 Isolation and quantification of RNA

Total RNA content was extracted from PCa‐exosomes using Exosomal RNA Isolation kits (NGB‐58000, Norgen Biotek) and from tissues and cells using MiRneasy kits (217004, QIAGEN). For miRNA expression, the complementary DNA (cDNA) of miRNA was synthesized using the TaqMan miRNA assay kit. Meanwhile, the cDNA of mRNA was synthesized using Transcriptor first‐strand cDNA synthesis kits. Thereafter, reverse‐transcription real‐time PCR (RT–qPCR) was performed using FastStart Universal SYBR Green Master Mix (Roche). The aforementioned mRNA primers and miRNA‐specific forward primer were provided by the Sangon Biotech, while the universal reverse primers were provided by the TaqMan miRNA assays kit, and miR‐205‐5p probe and primer were provided by TaqMan advanced miRNA assays. The expression patterns of U6 were detected using TaqMan RNU6B miRNA quantitative PCR kits (MTT01501, Biolab), and the cel‐miR‐39 was detected with TaqMan cel‐miR‐39 miRNA quantitative PCR kits (MTT01513, Biolab). Data analysis was performed using the 2^–ΔΔCt^ method. U6 worked as the loading control for miRNA, Glyceraldehyde‐3‐phosphate dehydrogenase (GAPDH) for other genes, and cel‐miR‐39 for miRNA in exosomes (Table S1).

### 2.13 Western blot analysis

Total protein content was extracted from the tissues and cells and lyzed in radio immunoprecipitation (RIPA) (P0013B, Beyotime) buffer containing protease inhibitor (A8260, Solarbio). The protein concentration was detected using a BCA kit (A53225, Thermo Fisher Scientific). Next, the obtained proteins underwent SDS‐PAGE separation, and electro‐transferring onto a poly(vinylidene fluoride) membrane (0.22 μm, Merck Millipore). The membrane underwent blocking with 5% skimmed milk powder at the ambient temperature for duration of 1 h and overnight incubation at 4°C with primary antibodies to GAPDH (#5174, CST), RUNX2 (#12556, CST), SFPQ (ab264197, Abcam), PTBP1 (#57246, CST), ALIX (ab88388, Abcam), TSG101 (ab125011, Abcam), CD9 (ab92726, Abcam), GRP94 (ab238126, Abcam), albumin (ab207327, Abcam), ALP (ab229126, Abcam), alpha‐1 type 1 collagen (COL1A1; ab34710, Abcam), osteocalcin (OCN; ab133612, Abcam), and Rab27a (#69295, CST). The following day, the membrane was subjected to 1‐h incubation with the peroxidase‐conjugated secondary antibody (goat anti‐rabbit immunoglobulin (IgG) (H+L; #111035003, acquired from Jackson ImmunoResearch). After three Tris‐buffered saline Tween rinses, the membrane was added with the luminescent solution (34094, Thermo Fisher Scientific) and developed in the Alliance Q9 Touch (UVITEC), with the results analyzed by Image J software. The ratio of the gray value of protein bands to that of internal reference (GAPDH) was deemed as the relative protein expression.

### 2.14 Luciferase reporter assay

The NEAT1 sequence containing miR‐205‐5p binding site and mutation binding site was cloned to the pmiRGlo vector to construct NEAT1‐wild type (WT) and NEAT1‐mutant (MUT) reporter vectors. Next, the reporter plasmids (RUNX2‐WT‐3′UTR and RUNX2‐MUT‐3′UTR) underwent co‐transfection with miR‐205‐5p mimic or mimic‐NC into the HEK‐293T cells (ATCC® CRL‐3216™). After 48 h, the cell supernatant was harvested, followed by luciferase activity measurement, which was expressed as the ratio of Firefly luciferase activity to Renilla luciferase activity. The binding ability of miR‐205‐5p to RUNX2 was detected using the same method.

### 2.15 Radioinmunoprecipitacion (RIP) assay

Magna RIP™ RNA‐Binding Protein Immunoprecipitation (IP) kits (Millipore) was used for this assay. Briefly, cell extracts underwent incubation with magnetic beads that had been conjugated with the Anti‐Ago2, anti‐IgG, or SFPQ antibody (Proteintech Group) at 4°C for 1 h. Next, the cell lysate was subjected to overnight incubation at 4°C with magnetic beads. Later, immunoprecipitated RNAs underwent purification and quantification by means of RT‐qPCR.

### 2.16 RNA pull‐down assay

PCa cells underwent lysing by RIPA lysis buffer, incubation at 37°C with Bio‐NC, and Bio‐NEAT1 for 1 h. Next, the samples were subjected to incubation at 37°C with Streptavidin agarose beads (Invitrogen) for 1 h. Finally, the eluants were collected in order to detect the expression patterns of miR‐205‐5p using RT‐qPCR.

### 2.17 **RNA‐**fluorescence ***in situ*** hybridization (**FISH**)

RNA‐FISH experiments were performed using RNA *in situ* hybridization fluorescence kits (Guangzhou Ribo Biotechnology Co., Ltd.) as per the manufacturer's instructions. Briefly, PCa cells were plated on glass slides, fixed with 4% PFA for 10 min and rinsed with PBS. Next, the fixed cells were placed in hybridization buffer containing 20 μM FISH probes and allowed to react overnight at 37°C. The following day, the slides were rinsed with PBS and re‐stained with the help of DAPI. Finally, the samples were observed under a confocal fluorescence microscope (Zeiss, LSM710). The probe for NEAT1 was designed and synthesized by Ribo Biotechnology.

### 2.18 IP assay

Cells were incubated in lysis buffer that comprised 100 mM portion of Tris‐HCl (pH 7.4, acquired from Sangon), 0.5% Triton X‐100 (acquired from Sangon), 150 mM portion of NaCl, 10% v/v glycerol, and protease inhibitor cocktail. Next, the obtained cell lysates were centrifuged, with the supernatant collected. Protein A/G sepharose beads were then dropped to the supernatant to pre‐clear nonspecific binding. Thereafter, the SFPQ antibody was supplemented and incubated with the pre‐cleared lysates at the controlled temperature of 4°C. Post overnight incubation, the cells were supplemented with protein A/G sepharose beads for 1 h. The pellets underwent four washes with lysis buffer and elution with the help of SDS‐PAGE sample buffer, which was followed by analysis by means of western blot using either the SFPQ or PTBP2 antibody.

### 2.19 Animal studies

Based on the previous study, human cancer cell lines are divided into two groups: osteoblastic phenotype‐ and osteolytic phenotype‐inducing cell lines. The osteoblastic phenotype‐inducing cancer cell lines include human PCa cell lines and represent the osteoblastic phenotype when the cells are implanted into the tibia of immunodeficient mice.^27^ To elucidate the effect of PCa with overexpression of NEAT1 on tumor bone microenvironment, osteoblastic MDA‐PCa‐2b cells were transplanted into BALB/cAJcl‐nu/nu mice (aged 4–6 weeks old, acquired from Beijing Vital River Laboratory Animal Technology Co., Ltd., Beijing, China) as previously described.^28^ Briefly, a total of 1 × 10^5^ cells mixed with Matrix gel were implanted into the dorsal skin flap on the skull of BALB/c female mice. Post 4‐week implantation, the mice were sacrificed and the bone lesions were examined. The R_mCT2 and TRI/FCS‐BON were used for micro‐CT analysis. Hematoxylin and eosin (H&E) and von Kossa staining assays were performed to observe the formation of the tumor bone environment. Additionally, in order to investigate the effect of hBMSCs with overexpressed NEAT1 on the osteogenic induction of PCa cells, hBMSCs, and osteoblastic C4‐2B cells were injected into the tibia at a ratio of 1:1. Bioluminescence was subsequently measured (RLU) longitudinally serving as an indicator of tumor growth. The study endpoint was pre‐determined as 1 × 10^6^ RLU. At the study endpoint (after 7 weeks), the mice were euthanized. The tumor‐bearing tibia was separated to analyze osteogenesis using high‐resolution μCT scanning. The morphology of the tibia was observed with the use of Masson's trichrome staining. The Inveon Research Workplace software was adopted to calculate bone volume/total volume (BV/TV) and bone mineral density (BMD). Ethics Committee of the First Affiliated Hospital, Sun Yat‐Sen University, approved the animal experiments.

### 2.20 Statistical analysis

Data analyses were performed using the SPSS 19.0 software (IBM). Measurement data were displayed as a form of mean ± standard deviation from at least three times of experiments. Data between two groups were compared by unpaired *t*‐test. The difference analysis among multiple groups was implemented by means of one‐way analysis of variance (ANOVA) and that at different time points was implemented by means of repeated measures ANOVA. Pairwise comparison was conducted using Tukey post hoc test. *p* < 0.05 was suggestive of statistically significant.

## 3 RESULTS

### 3.1 PCa‐exosomes promoted osteogenic differentiation of hBMSCs

**FIGURE 1:**
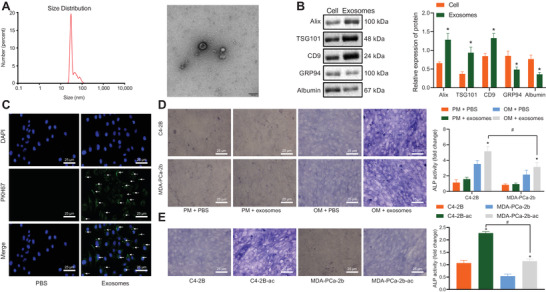
Prostate cancer (PCa)‐exosomes affected the osteogenic differentiation of human bone marrow‐derived mesenchymal stem cells (hBMSCs). (A) Diameter of C4‐2B cell derived‐exosomes observed under the transmission electron microscopy (bar = 100 nm) determined by nanoparticle tracking analysis (NTA). (B) The expression of ALIX, tumor suppressor gene 101, CD9, glucose‐regulated protein 94, and albumin in the exosomes was determined using western blot analysis as well as corresponding quantitative analysis. (C) PKH67‐labeled C4‐2B cell derived‐exosomes entered hBMSCs observed by immunofluorescence microscope. PKH67‐labeled C4‐2B cell derived‐exosomes were green, DAPI‐stained nucleus was blue (400 ×), and white arrows indicated green PKH67‐labeled PCa‐exosomes. (D) Detection of alkaline phosphatase (ALP) activity in hBMSCs treated with 50 μg/ml exosomes by ALP staining (400 ×). (E) Detection of ALP activity in hBMSCs treated with MDA‐PCa‐2b cell‐derived or C4‐2B cell‐derived exosomes cultured with 50 μg/ml normal medium or acid medium by ALP staining (400 ×). PM, proliferative medium; OM, osteogenic medium. In panel (B) and (D): *p < 0.05 versus cell/OM + PBS #*p* < 0.05 versus C4‐2B. In panel (E): *p < 0.05 versus C4‐2B/MDA‐PCa‐2b, #*p* < 0.05 versus C4‐2B‐ac. Data are shown as the mean ± standard deviation of three technical replicates. Unpaired *t*‐test was used for analysis of differences between two groups. Data among multiple groups were compared using one‐way ANOVA, and pairwise comparison was conducted using Tukey

Exosomes were isolated from PCa cells to investigate the interaction between PCa cells and hBMSCs and whether it was mediated by exosomes secreted by PCa cells. The results of TEM and NTA demonstrated that the exosomes of C4‐2B cells were bilayer vesicles, and the particles were in the range of 40–140 nm (Figure 1A). Meanwhile, western blot analysis revealed that the expression of ALIX, TSG101, and CD9 was detected in the exosomes, while the non‐exosomes markers (GRP94 and serum albumin) were significantly less (Figure 1B). These data indicated that the C4‐2B cell‐derived exosomes were successfully isolated. To further explore the effect of C4‐2B cell‐derived exosomes on hBMSCs, PKH67‐labeled C4‐2B cell‐derived exosomes were co‐cultured with hBMSCs for 12 h. Subsequently, it was found that a large number of C4‐2B cell‐derived exosomes entered the hBMSCs and were distributed around the nucleus (Figure 1C), indicating that C4‐2B cell‐derived exosomes can enter the cells. Then, hBMSCs were treated with exosomes isolated from MDA‐PCa‐2 and C4‐2B to observe the osteogenesis of hBMSCs. After 7 days of osteogenic stimulation, ALP staining and activity of hBMSCs treated with C4‐2B‐derived exosomes and MDA‐PCa‐2b‐derived exosomes were found to be significantly higher than those in untreated hBMSCs, while the ALP staining and activity were significantly lower in hBMSCs treated with exosomes with MDA‐PCa‐2b than with C4‐2B (Figure 1D). In addition, the tumor microenvironment *in vivo* is an acidic environment. In order to better correspond the experimental results *in vitro* to *in vivo*, we further used an acidic medium (pH 6.5) to culture MDA‐PCa‐2b (MDA‐PCa‐2b‐ac) as well as C4‐2B (C4‐2B‐ac) and then isolated exosomes. After treatment of hBMSCs, osteogenesis of hBMSCs was observed. We found that after 7 days of osteogenic stimulation, the ALP staining and activity of hBMSCs treated with C4‐2B‐ac‐derived exosomes were significantly higher than those of hBMSCs treated with C4‐2B‐derived exosomes, while the ALP staining and activity of hBMSCs treated with MDA‐PCa‐2b‐ac‐derived exosomes were not significantly increased as those of hBMSCs treated with C4‐2B‐ac‐derived exosomes (Figure 1E). These findings suggested that exosomes could initiate the osteogenic differentiation potentials of hBMSCs, and the osteogenic effect of exosomes from high metastatic PCa cells was more obvious, so was the osteogenesis of exosomes from cancer cells under acidic culture condition.

### 3.2 PCa‐exosomes enhanced NEAT1 expression in hBMSCs

**FIGURE 2:**
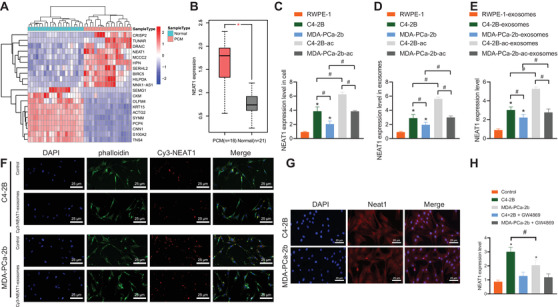
Nuclear‐enriched abundant transcript 1 (NEAT1) could be transferred into hBMSCs via PCa‐derived exosomes. (A) Unsupervised clustering heat map of the first 10 DEGs. Each row represents a gene and each column represents a sample. PMC represents metastatic PCa group and Normal represents normal prostate group. (B) The expression of NEAT1 is shown in the box diagram. Red indicates metastatic PCa group, and gray indicates normal prostate group. (C) NEAT1 expression in RWPE‐1 cells and MDA‐PCa‐2b and C4‐2B cells cultured in normal medium and acid medium measured by RT‐qPCR. (D) NEAT1 expression in exosomes derived from RWPE‐1 cells and MDA‐PCa‐2b and C4‐2B cells cultured in normal medium and acid medium measured by RT‐qPCR. (E) NEAT1 expression in hBMSCs co‐cultured with exosomes derived from RWPE‐1 cells as well as MDA‐PCa‐2b and C4‐2B cells cultured in normal medium and acid medium measured by RT‐qPCR. *p < 0.05 versus RWPE‐1‐exosomes; #*p* < 0.05. (F) The uptake of cy3‐NEAT1‐labeled PCa‐exosomes by MSCs observed under the fluorescence microscope (400 ×). Cy3‐NEAT1‐labeled exosomes were red, DAPI‐stained nuclei were blue, and phalloidin‐labeled MSCs were green. (G) The localization of NEAT1 in hBMSCs co‐cultured with PCa‐exosomes determined by RNA‐FISH, wherein NEAT1 was red and DAPI‐stained nucleus was blue (400 ×). (H) NEAT1 expression in hBMSCs co‐cultured with PCa cells and treated with GW4869. #*p* < 0.05; *p < 0.05 versus normal prostate group, RWPE‐1 cells, PBS group, or RWPE‐1‐exosomes/control. Data are shown as the mean ± standard deviation of three technical replicates. Data among multiple groups were compared using one‐way ANOVA, and pairwise comparison was conducted using Tukey

The “limma” software package of R language was employed to analyze the DEGs to plot an unsupervised cluster heat map of the top 10 DEGs. It was found that NEAT1 was in the top 10 upregulated genes in metastatic PCa (Figure 2A), and the box diagram was used to show the expression patterns of NEAT1 in metastatic PCa and normal prostate tissues (Figure 2B), wherein NEAT1 was highly expressed in metastatic PCa. In addition, NEAT1 is reported to be present in exosomes and numerous types of cells (http://www.EVsrbase.org). As a result, the current study further investigated the presence of NEAT1 in PCa‐exosomes and the effect of NEAT1 on hBMSCs. The results of RT‐qPCR displayed that NEAT1 expression levels were upregulated in C4‐2B cells relative to RWPE‐1 cells, while the differences were not evident in MDA‐PCa‐2b cells, and NEAT1 expression was the highest in C4‐2B cultured by acid medium (pH 6.5; C4‐2B‐ac; Figure 2C). Similar results were observed in PCa‐exosomes (Figure 2D). Subsequently, hBMSCs were co‐cultured with RWPE‐1‐exosomes and PCa‐exosomes followed by determination of the NEAT1 expression patterns. Compared with RWPE‐1‐exosomes, both PCa‐exosomes significantly elevated NEAT1 expression levels in hBMSCs, especially in C4‐2B‐derived exosomes, and increase in NEAT1 expression was the most significant in C4‐2B‐ac‐derived exosomes (Figure 2E). Therefore, it was speculated that PCa‐exosomes may affect the osteogenic differentiation of hBMSCs by transferring NEATI. Subsequently, in order to confirm that PCa cells could transfer NEATI to hBMSCs through exosomes, we transfected PCa with Cy3‐labeled NEATI. The supernatant was harvested after 48 h for the ultracentrifugation purpose to obtain exosomes, which were thereafter co‐cultured with MSCs for 12 h and fixed (Figure 2F). The results demonstrated the presence of Cy3‐labeled NEAT1 in the cytoplasm of MSCs. Additionally, NEAT1 localization was examined using RNA‐FISH, which also revealed that NEAT1 uptaken by hBMSCs was localized in the cytoplasm (Figure 2G). Meanwhile, RT‐qPCR exhibited that NEAT1 expression levels were upregulated in hBMSCs co‐cultured with PCa cells, while the secretion of exosomes was inhibited in PCa cells treated with GW4869, and NEAT1 expression showed no significant difference (Figure 2H). Altogether, these findings indicated that NEAT1 in PCa could be transferred to hBMSCs via exosomes.

### 3.3 NEAT1 shuttled by PCa‐exosomes promoted osteogenic differentiation potentials of hBMSCs

**FIGURE 3:**
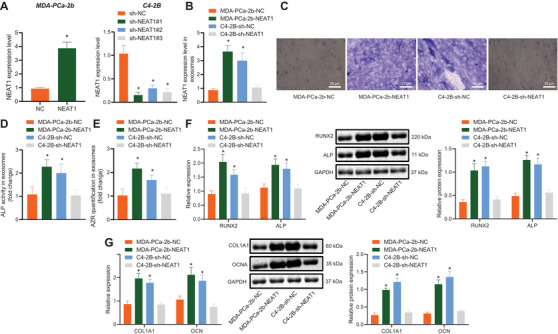
PCa‐exosomes carrying NEAT1 accelerated osteogenic differentiation of hBMSCs. MDA‐PCa‐2b cells were treated with exogenous overexpressed NEAT1, and C4‐2B cells were treated with shRNA targeting NEAT1. (A) NEAT1 expression in MDA‐PCa‐2b and C4‐2B cells determined by RT‐qPCR. The shRNA (sh‐NEAT1#1) with best efficiency was selected for subsequent experiments. (B) NEAT1 expression in exosomes derived from MDA‐PCa‐2b and C4‐2B cells determined by RT‐qPCR. hBMSCs were co‐cultured with 50 μg/ml exosomes. (C) Osteogenic differentiation was detected by ALP staining on the seventh day and alizarin red staining (ARS) on the 14th day (400 ×). (D) ALP activity of hBMSCs in PM and OM on the seventh day was detected. (E) The ARS mineralization of hBMSCs in PM and OM on the 14th day was determined. (F) The mRNA and protein levels of runt‐related transcription factor 2 (RUNX2) and ALP in PM and OM on the seventh day determined by RT‐qPCR and western blot analysis. (G) The mRNA and protein levels of alpha‐1 type 1 collagen (COL1A1) and osteocalcin (OCN) in PM and OM on the 14th day determined by RT‐qPCR and western blot analysis. *p < 0.05 versus NC, sh‐NC, or MDA‐PCa‐2b‐NC group. Data are shown as the mean ± standard deviation of three technical replicates. Unpaired *t*‐test was used for analysis of differences between two groups. Data among multiple groups were compared using one‐way ANOVA, and pairwise comparison was conducted using Tukey. PM, proliferation medium; PTBP2, polypyrimidine tract‐binding protein 2

To further determine the function of NEAT1, we performed GSEA analysis using GO_BP as a background and found that NEAT1 expression was significantly enriched on 19 items, and these GO_BP items were primarily related to DNA replication and metabolism (Figure 3A, Table S2). Meanwhile, various studies have highlighted that DNA replication and cell metabolism can confer effects on osteogenic differentiation,^29^, ^30^ whereas NEAT1 is known to promote the osteogenic differentiation of stem cells.^15^ Consequently, in order to verify whether the upregulation of NEAT1 in hBMSCs could promote osteogenic differentiation, we treated hBMSCs with exogenous overexpressed NEAT1 (Figure S1A). It was found that overexpressed NEAT1 did not alter the proliferation ability of hBMSCs (Figure S1B) but significantly promoted the ALP staining and activity of hBMSCs after osteogenic stimulation. In addition, ARS staining along with quantification on Day 14 also illustrated that the mineralization of extracellular matrix increased significantly after the restoration of NEAT1 (Figures S1C,D). On the other hand, after exogenous NEAT1 stimulated the osteogenic induction of hBMSCs, the mRNA levels of ALP, COL1A1, RUNX2, and OCN were found to be markedly upregulated (Figures S1E,F). Similarly, to verify that NEAT1 exhibited the same effect on hBMSCs in PCa‐exosomes, exosomes with high or poor NEAT1 expressions were extracted from MDA‐PCa‐2b and C4‐2B cells treated with overexpression or knockdown of NEAT1, respectively (Figures 3B,C). The ALP staining, activity, and mineralization of extracellular matrix of hBMSCs treated with overexpressed NEAT1 (MDA‐PCa‐2b‐NEAT1‐exosomes and C4‐2B‐sh‐NC‐exosomes) were all found to be significantly higher than that of hBMSCs with downregulated NEAT1 (MDA‐PCa‐2b‐NC‐exosomes and C4‐2B‐sh‐NEAT1‐exosomes; Figures 3D–F). Moreover, the mRNA and protein levels of RUNX2, ALP, COL1A1, and OCN were also highly expressed in PCa‐exosomes with elevated NEAT1 (Figures 3G,H). To conclude, these findings suggested that PCa‐exosomes‐transferred NEAT1 enhanced the osteogenic differentiation of hBMSCs.

### 3.4 PCa‐exosomes delivered NEAT1 into hBMSCs to upregulate RUNX2 expression by means of competitively binding to miR‐205‐5p

**FIGURE 4:**
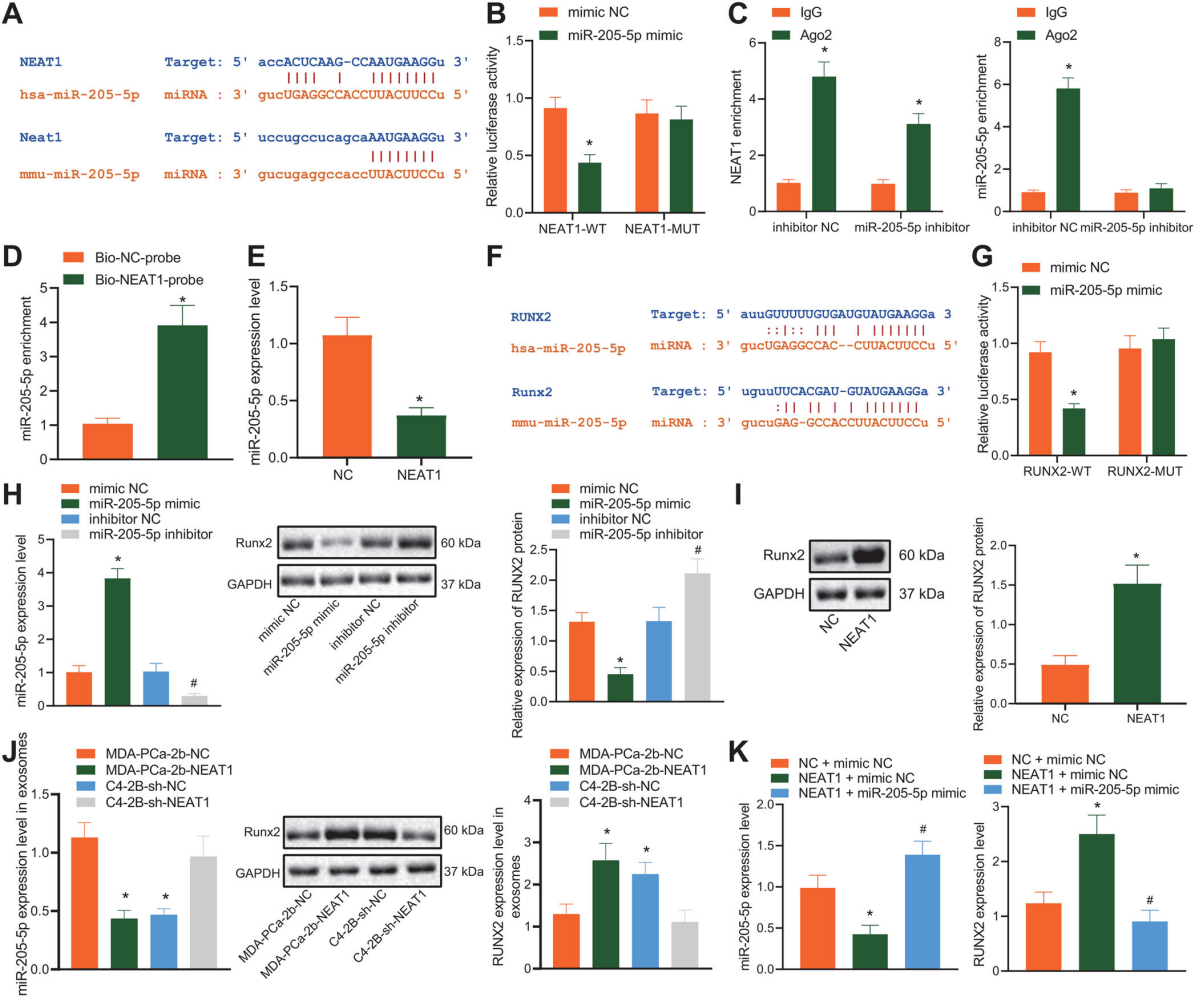
NEAT1 elevated RUNX2 expression by competitively binding to miR‐205‐5p. (A) the complementary binding sites between NEAT1 and miR‐205‐5p in the StarBase website. (B) Luciferase activity of NEAT1‐WT/MUT detected by dual‐luciferase reporter gene assay. (C) Enrichment of NEAT1 and miR‐205‐5p by Anti‐Ago2 or Anti‐IgG detected by RNA immunoprecipitation (RIP) assay. (D) Enrichment of miR‐205‐5p detected by RNA pull‐down assay. hBMSCs were treated with exogenous overexpressed NEAT1. (E) miR‐205‐5p expression in hBMSCs determined by RT‐qPCR. (F) A targeted binding site between miR‐205‐5p and RUNX2 predicted by StarBase. (G) Luciferase activity of WT/MUT‐RUNX2 detected by dual‐luciferase reporter gene assay. hBMSCs were transfected with miR‐205‐5p mimic (mimic NC) or miR‐205‐5p inhibitor (inhibitor NC). (H) RUNX2 expression in hBMSCs determined by RT‐qPCR and western blot analysis as well as corresponding quantitative analysis. (I) RUNX2 expression in hBMSCs treated with overexpressed NEAT1 detected by western blot analysis as well as corresponding quantitative analysis. (J) RUNX2 expression in hBMSCs co‐cultured with PCa‐exosomes with high or poor NEAT1 expression detected by western blot analysis. (K) miR‐205‐5p and RUNX2 expression in MSCs treated with restored NEAT1 and miR‐205‐5p mimic determined by RT‐qPCR and western blot analysis, respectively. *p < 0.05 versus mimic NC, IgG, Bio‐NC‐probe, MDA‐PCa‐2b‐NC, or NC + mimic NC group. Data are shown as the mean ± standard deviation of three technical replicates. Unpaired *t*‐test was used for analysis of differences between two groups. Data among multiple groups were compared using one‐way ANOVA, and pairwise comparison was conducted using Tukey

The StarBase website predicted the presence of complementary binding sites between NEAT1 and miR‐205‐5p (Figure 4A). Subsequently, dual‐luciferase reporter gene, RNA IP (RIP), and RNA pull‐down assays were performed to explore whether NEAT1 acts as ceRNA to alter the miR‐205‐5p expression in hBMSCs. The results obtained from the dual‐luciferase reporter gene assay exhibited that luciferase activity of NEAT1‐WT was inhibited in the presence of miR‐205‐5p mimic, while no evident differences was observed in NEAT1‐MUT (Figure 4B). Meanwhile, RIP assay showed that NEAT1 was present in the Ago2 immunoprecipitate but decreased significantly in the purified Ago2 complex from the cells treated with miR‐205‐5p inhibitor (Figure 4C), indicating that NEAT1 may exist in the miR‐205‐5p RISC complex. Furthermore, RNA pull‐down assay demonstrated that there were a large number of miR‐205‐5p in NEAT1 pull‐down pellets (Figure 4D), suggesting that miR‐205‐5p had sequence specificity for NEAT1 recognition. Meanwhile, in hBMSCs with upregulated NEAT1, miR‐205‐5p expression was found to be significantly decreased (Figure 4E). The aforesaid data suggested that NEAT1 could competitively bind to miR‐205‐5p.

The aforementioned results indicated that NEAT1 may act as a ceRNA to modulate the target gene of miR‐205‐5p. Existing evidence suggests that the lineage process of osteoblasts and chondrocytes is strictly controlled by the transcription factor RUNX2.^31^ Moreover, the Starbase bioinformatics website indicated a targeted binding site between miR‐205‐5p and RUNX2 (Figure 4F). Dual‐luciferase reporter gene assay data further demonstrated that luciferase activity of WT‐RUNX2 was inhibited in the presence of miR‐205‐5p mimic, while no evident differences were found in MUT‐RUNX2 (Figure 4G). RT‐qPCR and western blot analysis data exhibited that RUNX2 expression was reduced in hBMSCs overexpressing miR‐205‐5p while contrary results were obvious in the hBMSCs treated with exogenous miR‐205‐5p inhibitor (Figure 4H). Interestingly, when hBMSCs were treated with overexpressed NEAT1 or PCa‐exosomes, both NEAT1 and NEAT1 shuttled by exosomes (MDA‐PCa‐2b‐NEAT1‐exosomes and C4‐2B‐sh‐NC‐exosomes) were found to significantly promote the RUNX2 expression (Figures 4I,J). However, hBMSCs treated with restored NEAT1 and miR‐205‐5p mimic could inhibit the upregulation of RUNX2 induced by NEAT1 (Figure 4K). These findings suggested that NEAT1 could act as a ceRNA of miR‐205‐5p, resulting in increased expressions of RUNX2, a miR‐205‐5p target gene.

### 3.5 **NEAT1 promoted RUNX2** expression in hBMSCs **via** PTBP2/SFPQ

**FIGURE 5:**
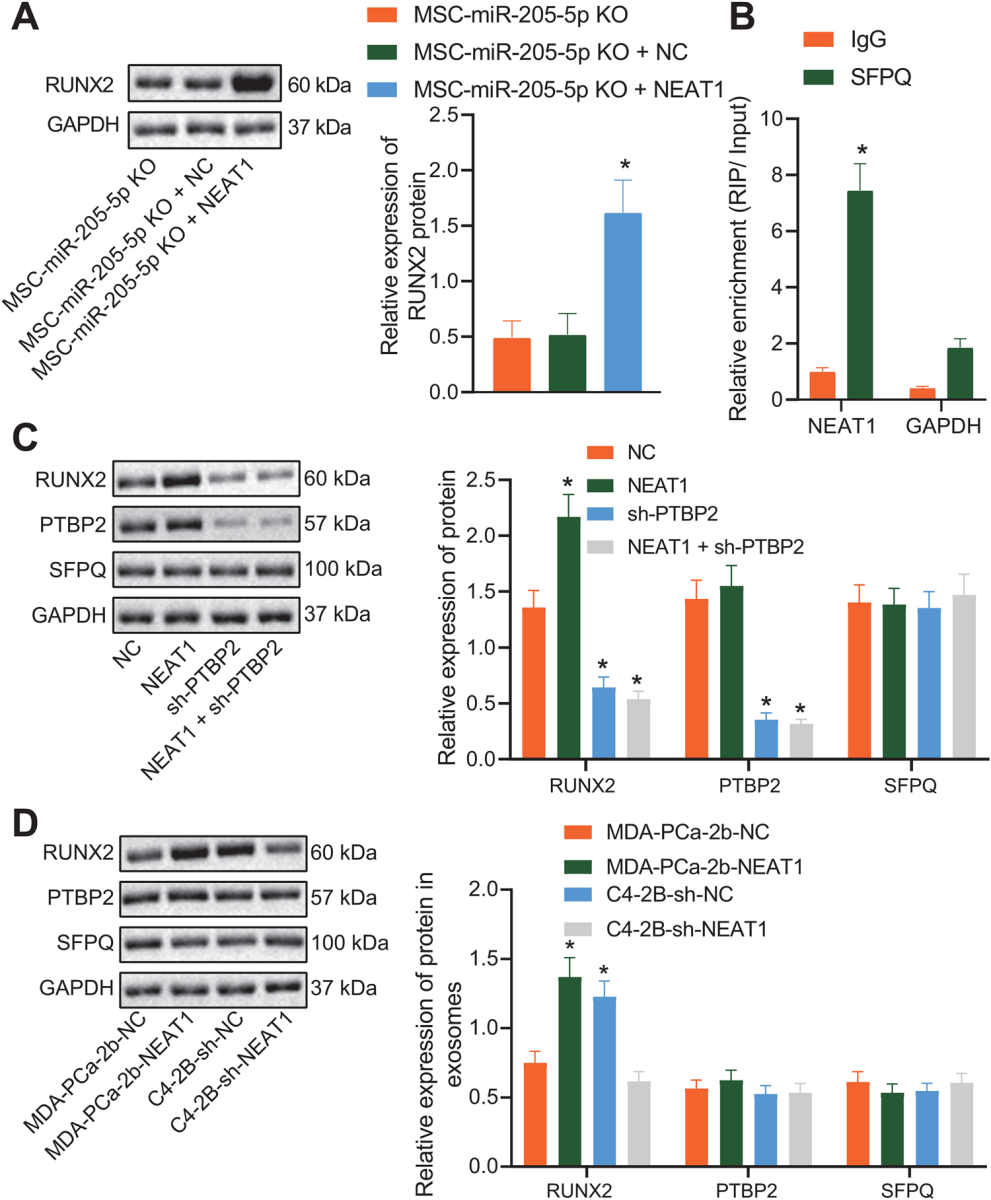
NEAT1 influenced splicing factor proline‐ and glutamine‐rich (SFPQ)/PTBP to regulate RUNX2 expression. hBMSCs with miR‐205‐5p knockout were treated with NEAT1 overexpression. (A) RUNX2 expression in hBMSCs detected by western blot analysis as well as corresponding quantitative analysis. (B) Interaction between NEAT1 and SFPQ in hBMSCs detected by RIP assay. (C) Interaction between NEAT1 and SFPQ and RUNX expressions in hBMSCs treated with upregulated NEAT1 and PTBP2 knockdown detected by western blot analysis. (D) Interaction between NEAT1 and SFPQ and RUNX expressions in hBMSCs treated with PCa‐exosomes with NEAT1 overexpression detected by western blot analysis. *p < 0.05 versus MSC‐miR‐205‐5p KO, IgG, NC, or MDA‐PCa‐2b‐NC group. Data are shown as the mean ± standard deviation of three technical replicates. Unpaired *t*‐test was used for analysis differences between two groups. Data among multiple groups were compared using one‐way ANOVA, and pairwise comparison was conducted using Tukey

Published data have reported that NEAT1 induction can promote the transcriptional activation of IL8 by relocating SFPQ to the paraspeckles from the IL8 promoter.^32^ Moreover, MALAT1 is known to bind to SFPQ and release free PTBP2 by dissociating the SFPQ/PTBP2 dimer, thereby elevating the translational levels of RUNX2 through interaction with IRES domain in the 5′UTR of the corresponding RUNX2 mRNAs.^24^ As a result, we explored whether NEAT1 promoted RUNX2 expression in hBMSCs through PTBP2/SFPQ axis in a similar manner. hBMSCs with miR‐205‐5p knockout were treated with NEAT1 overexpression and RUNX2 expression was also significantly upregulated (Figure 5A). Therefore, we speculated whether NEAT1 could also upregulate RUNX2. RIP assay was then performed in order to determine whether hBMSCs can affect the expression levels of RUNX2 by SFPQ/PTBP2 complex through NEAT1, which revealed that NEAT1 interacted with SFPQ in hBMSCs (Figure 5B), which indicated that NEAT1 could combine with SFPQ to affect the function of SFPQ. Similarly, western blot analysis exhibited that knockdown of PTBP2 weakened the ability of NEAT1 to increase RUNX2 expression levels (Figure 5C). Moreover, RUNX2 expression was found to be elevated in hBMSCs treated with PCa‐exosomes with NEAT1 overexpression (Figure 5D). Together, NEAT1 could promote the expression of RUNX2 in hBMSCs by regulating the PTBP2/SFPQ complex.

### 3.6 NEAT1 shuttled by PCa‐exosomes promoted the osteogenic differentiation potentials of hBMSCs by regulating RUNX2

**FIGURE 6:**
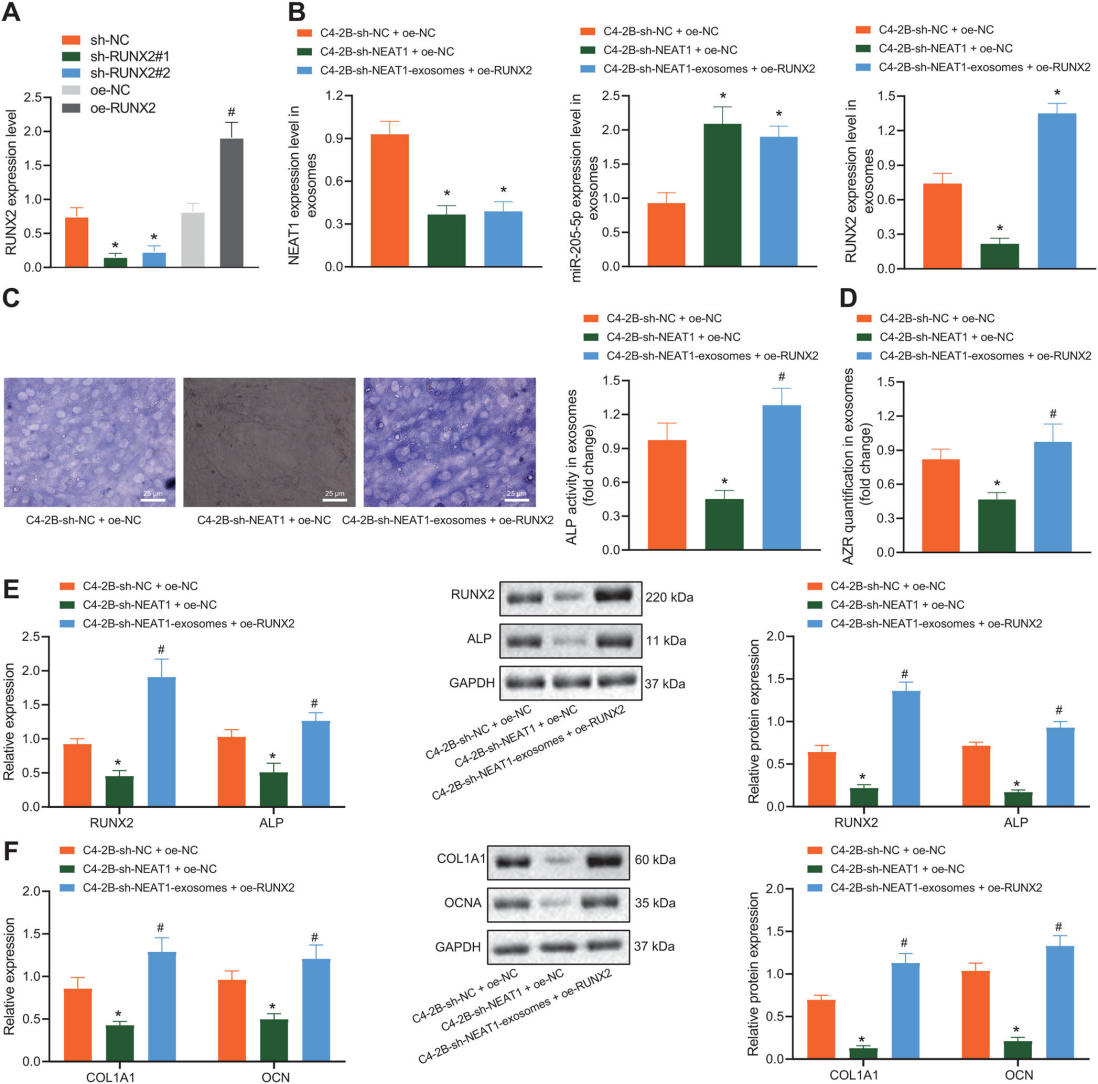
NEAT1 shuttled by C4‐2B‐derived exosomes affected osteogenic differentiation of hBMSCs by regulating RUNX2. hBMSCs were treated with sh‐RUNX2 or oe‐RUNX2. (A) RUNX2 expression in hBMSCs determined by western blot analysis. hBMSCs were treated with upregulated RUNX2 and C4‐2B‐derived exosomes with NEAT1 knockdown. (B) Expression of NEAT1 and miR‐205‐5p and RUNX2 protein level were assessed by RT‐qPCR and western blot analysis. (C) ALP activity of hBMSCs was detected by ALP staining on the seventh day (400 ×). (D) The ARS mineralization of hBMSCs was detected by ARS staining on the 14th day. (E) The mRNA and protein levels of RUNX2 and ALP determined by RT‐qPCR and western blot analysis on the seventh day. (F) The mRNA and protein levels of COL1A1 and OCN determined by RT‐qPCR and western blot analysis on the 14th day. *p < 0.05 versus MDA‐PCa‐2b‐NC + sh‐NC group. #*p* < 0.05 versus MDA‐PCa‐2b‐NEAT1 + sh‐NC group. Data are shown as the mean ± standard deviation of three technical replicates. Unpaired *t*‐test was used for analysis differences between two groups. Data among multiple groups were compared using one‐way ANOVA, and pairwise comparison was conducted using Tukey

To further explore the effects of the NEAT1/RUNX2 axis on the osteogenic differentiation potentials of hBMSCs, RUNX2 was overexpressed or knocked down (Figure 6A). Next, we treated hBMSCs with decreased RUNX2 and MDA‐PCa‐2b‐derived exosomes with NEAT1 overexpression (MDA‐PCa‐2b‐NEAT1‐exosomes) and also treated hBMSCs with upregulated RUNX2 and C4‐2B‐derived exosomes with NEAT1 knockdown (C4‐2B‐sh‐NEAT1‐exosomes). Compared with MDA‐PCa‐2b‐NC‐exosomes + sh‐NC, NEAT1 and RUNX2 expressions were elevated and that of miR‐205‐5p was reduced in hBMSCs treated with NEAT1 overexpression shuttled by MDA‐PCa‐2b‐derived exosomes, whereas versus the MDA‐PCa‐2b‐NEAT1‐exosomes + sh‐NC, the expression of NEAT1 and miR‐205‐5p showed no obvious differences, while that of RUNX2 was downregulated in MSCs treated with silenced RUNX2 and NEAT1 overexpression shuttled by MDA‐PCa‐2b‐derived exosomes (Figure S2A). Compared with C4‐2B‐sh‐NC‐exosomes + oe‐NC, NEAT1 and RUNX2 expressions were decreased and that of miR‐205‐5p was increased in hBMSCs treated with NEAT1 knockdown shuttled by C4‐2B‐derived exosomes, whereas compared with C4‐2B‐sh‐NEAT1‐exosomes + oe‐NC, the expression of NEAT1 and miR‐205‐5p showed no obvious differences and that of RUNX2 was upregulated in hBMSCs treated with restored RUNX2 and NEAT1 downregulation shuttled by C4‐2B‐derived exosomes (Figure 6B). Furthermore, the results of osteogenic differentiation assay revealed that, compared with MDA‐PCa‐2b‐NC‐exosomes + sh‐NC, extracellular matrix mineralization, ALP staining, and activity of MSCs were significantly increased, and the mRNA and protein levels of RUNX2, ALP, COL1A1, and OCN were also promoted in the MDA‐PCa‐2b‐NEAT1‐exosomes + sh‐NC group. However, when RUNX2 expression was knocked down, the promotive effect of exosomes with high NEAT1 expression on osteogenic differentiation of hBMSCs was found to be weakened (Figures S2B–E). Meanwhile, compared with C4‐2B‐sh‐NC‐exosomes + oe‐NC, ALP staining, activity, and extracellular matrix mineralization of MSCs were all decreased, and the mRNA and protein levels of RUNX2, ALP, COL1A1, and OCN were also repressed. Whereas, compared with C4‐2B‐sh‐NEAT1‐exosomes + oe‐NC, osteogenic differentiation of hBMSCs was found to be induced in MSCs treated with restored RUNX2 and NEAT1 downregulation shuttled by C4‐2B‐derived exosomes (Figures 6C–F). These findings supported that NEAT1 carried by exosomes could promote the RUNX2 expression and osteogenic differentiation of bBMSCs.

### 3.7 Upregulated NEAT1 induced osteogenesis *in vivo*

**FIGURE 7:**
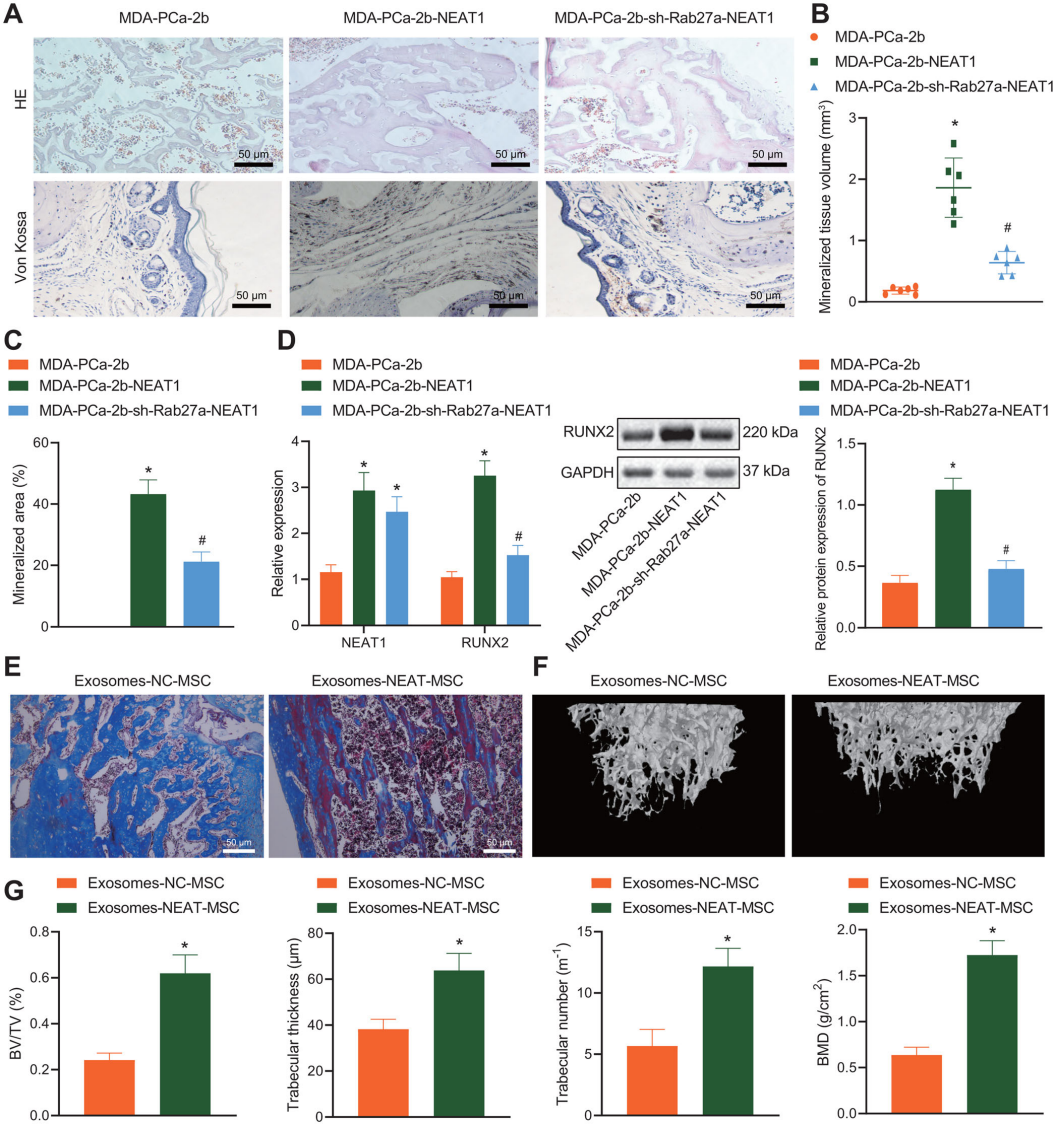
Overexpression of NEAT1 induced osteogenesis *in vivo*. (A) Osteoblast lesions observed using H&E staining and von Kossa staining (200 ×). (B) The volume of mineralized tissues was quantified. (C) The proportion of mineralized bone matrix was quantified. (D) The expression of NEAT1 and RUNX2 mRNA in tumor tissues determined by RT‐qPCR and the protein expression of RUNX2 determined by western blot analysis. (E) The osteogenic induction of PCa cells observed using Masson's trichrome staining (50 μm). (F) Representative μCT images of tibia. (G) According to the typical μCT images of tibia after 7 weeks of inoculation, bone volume as function of total volume, trabecular thickness, trabecular number, and bone mineral density were calculated with μCT value. *p < 0.05 versus MDA‐PCa‐2b or exosomes‐NC‐MSC group. #*p* < 0.05 versus MDA‐PCa‐2b‐NEAT1 group. *N* = 6. Data are shown as the mean ± standard deviation of three technical replicates. Unpaired *t*‐test was used for analysis differences between two groups. Data among multiple groups were compared using one‐way ANOVA, and pairwise comparison was conducted using Tukey

Last, we cloned MDA‐PCa‐2b‐NEAT1 cells to the skulls of immunodeficient mice to investigate whether NEAT1 in PCa‐exosomes can initiate osteoblastic phenotype in the bone metastatic microenvironment *in vivo*. It was found that the mineralized tissues presented bone matrix, surrounded by osteoblast‐like cells or osteoid cells (Figure 7A). Moreover, RUNX2 expression was markedly elevated in tumor tissues formed by MDA‐PCa‐2b cells overexpressing NEAT1. To further confirm that this change was mediated by the transfer of NEAT1 by exosomes, we knocked down Rab27A in MDA‐PCa‐2b cells, which regulates exosome secretion, and then implanted these MDA‐PCa‐2b cells into the skulls of immunodeficient mice (Figure S3) to inhibit the secretion of exosomes.^33^ We found that the effect of NEAT1 overexpression on bone metastatic microenvironment was significantly suppressed (Figures 7B–D). These findings concluded that NEAT1 shuttled by PCa cells through exosomes induced the osteogenic ability of peripheral cells.

Additionally, to further verify the effect of NEAT1 upregulation in hBMSCs on PCa‐induced osteogenesis, we employed C4‐2B models of osteogenic metastatic PCa to detect osteogenesis. In immunodeficient mice, cells were injected with cells at the ratio of 1:1 (C4‐2B: exosomes‐NC‐MSC (*n* = 6) or C4‐2B: exosomes‐NEAT1‐MSC (*n* = 6; Figure S4). Tumor‐bearing tibias were isolated after 7 weeks. The results of Masson's trichrome staining illustrated the osteogenesis induced by PCa‐exosomes with NEAT1 overexpression was facilitated (Figure 7E). Further analysis of osteogenesis by high‐resolution μCT scanning also revealed that PCa‐exosomes with NEAT1 overexpression significantly increased the PCa‐induced osteogenesis (Figure 7F), BV/TV, number, the thickness of bone trabeculae, and BMD (Figure 7G). Altogether, these findings indicated that hBMSCs treated with exosomes overexpressing NEAT1 enhanced the osteogenic induction of PCa cells.

## 4 DISCUSSION

Bone metastasis is still the main clinical problem in patients with advanced PCa.^34^ In the metastatic lesions, tumor cells are known to interact with various cell types (osteoblasts, osteoclasts, and MSCs), leading to an osteoblastic phenotype.^35^ Bone metastatic PCa results in extensive osteogenesis by inducing the MSC recruitment and osteoblastic differentiation potentials.^36^ As the specific mechanisms that affect the predominantly osteoblastic phenotype remain to be excavated, the current study sought to dissect out the effect of exosomes‐encapsulated NEAT1 from PCa cells, miR‐205‐5p, RUNX2, and SFPQ/PTBP2 in the osteogenic differentiation of hBMSCs and their underlying mechanisms. Our obtained findings suggested that NEAT1 shuttled by PCa‐derived exosomes could be transferred into hBMSCs, where NEAT1 exerted inductive effects on osteogenic differentiation of hBMSCs through the upregulation of RUNX2 by competitively binding to miR‐205‐5p and regulating SFPQ/PTBP2 both *in vitro* and *in vivo*.

First, findings obtained in our study revealed that exosomes derived from PCa could promote the osteogenic differentiation potentials of hBMSCs. In addition to that, we also found that NEAT1 from PCa cells transferred into hBMSCs via exosomes to induce the osteogenic differentiation of hBMSCs. Existing evidence suggests that exosomes are closely related to numerous processes of tumor development, such as tumor cell proliferation,^37^ cancer metastasis,^38^ and immune regulation.^39^ Of note, a previous research has reported the involvement of exosomes derived from tumor cells in the regulation of bone metastasis.^40^ Besides, PCa‐derived exosomes have been demonstrated to regulate osteoblast activity in the bone metastatic niche, mainly due to their cargo content in miRNAs, and they also inhibit osteoclast differentiation.^41^ Literature has also shown that NEAT1 can augment tumorigenesis by regulating cancer‐favorable transcriptome.^42^ Moreover, another study reported that NEAT1 knockdown inhibits the proliferation and tumorigenesis of PCa cells, which is in line with our findings.^43^ Furthermore, NEAT1 was recently highlighted to induce the osteogenic differentiation in hBMSCs in another report.^15^ Our findings in conjunction with existing data support that NEAT1 shuttled by exosomes from PCa cells could enhance the osteogenic differentiation potentials of hBMSCs.

Additionally, we uncovered that NEAT1 functions as a ceRNA of miR‐205‐5p. This in in accordance with the study performed by Liu et al., wherein NEAT1 was highlighted to sponge miR‐205‐5p to influence the progression of colorectal cancer.^44^ Furthermore, accumulating evidence has attributed great importance to the roles of miRNAs in osteogenic differentiation and bone development.^45^ More importantly, previous data have also documented that miR‐205‐5p works as an anti‐tumor miRNA to regulate the progression of PCa.^19^ Meanwhile, it is also noteworthy that miR‐205‐5p bound by lncRNA ENST00000563492 has been shown to facilitate the osteogenesis in BMSCs.^46^ Furthermore, our experimental findings demonstrated that NEAT1 inhibited miR‐205‐5p to promote the activity of ALP and mineralization of extracellular matrix and continuously upregulated the mRNA levels of RUNX2, ALP, COL1A1, and OCN, thereby contributing to osteogenic differentiation of hBMSCs and presenting a promising target for therapeutics through this axis.

Furthermore, our findings also confirmed that miR‐205‐5p targeted RUNX2. In accordance with our data, another study highlighted that RUNX2 could be targeted by miR‐205 to influence the metastasis of PCa.^31^ In addition, our study further demonstrated that NEAT1 shuttled by PCa‐exosomes promoted osteogenic differentiation potentials of hBMSCs by regulating RUNX2. Of note, RUNX2 is regarded as a transcription factor that is responsible for osteoblast differentiation.^47^ Meanwhile, the study performed by Senbanjo et al. illustrated that RUNX2 expression levels were elevated in PCa cells that metastasize to bone.^48^ Moreover, we discovered that NEAT1 could promote the expression of RUNX2 via the SFPQ/PTBP2 axis. NEAT1 is a scaffold for Drosophila behavior and RNA‐binding protein enrichment in the human splicing (DBHS) family, including SFPQ.^49^ Also, the interaction between PTBP2 and SFPQ regulated by MALAT‐1 RNA is known to promote tumor growth and metastasis.^50^ However, only a few studies have explored the underlying mechanisms of NEAT1 in RUNX2 upregulation via modulation of the SFPQ/PTBP2 axis to induce the osteogenic differentiation of hBMSCs.

**FIGURE 8:**
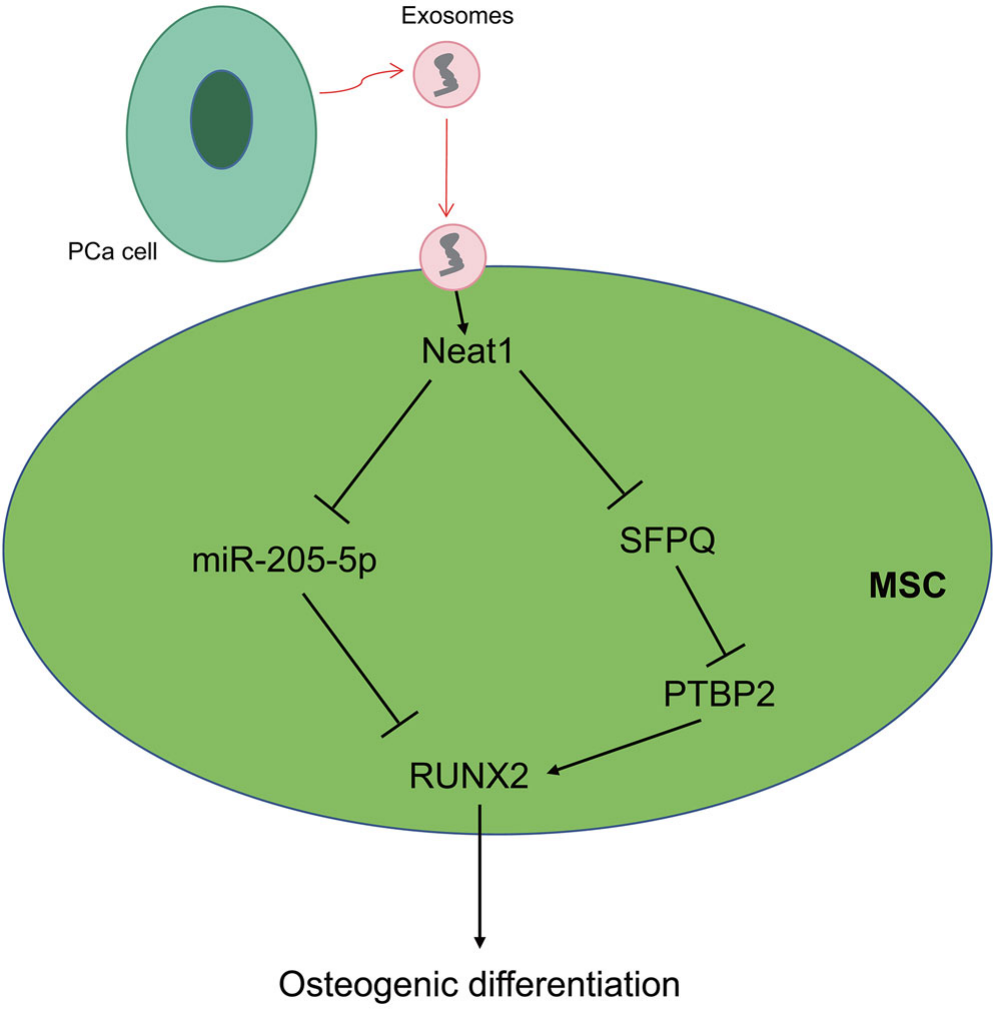
Schematic diagram showing the potential mechanism of NEAT1‐mediated effects on osteogenic differentiation of hBMSCs in PCa. PCa cells secrete exosomes containing NEAT1. After the internalization of exosomes by hBMSCs, NEAT1 promotes the osteogenic differentiation of hBMSCs through two mechanisms: first, NEAT1 acts as a ceRNA to bind to miR‐205‐5p and to upregulate RUNX2, the downstream target gene of miR‐205‐5p; second, NEAT1 binds to SFPQ to release PTBP2 from the SFPQ/PTBP2 complex. PTBP2 interacts with IRES domain of RUNX2 mRNA 5 'UTR to improve the translation level of RUNX2

Overall, the current study proved that transfer of NEAT1 via PCa‐derived exosomes altered the RUNX2 expression, by means of competitively binding to miR‐205‐5p and regulating the SFPQ/PTBP2 axis, consequently, leading to the osteogenic differentiation of hBMSCs (Figure 8). Our novel findings pave the way for the development of efficient therapeutic strategies to combat bone metastasis in PCa. Due to the limited data, the roles of NEAT1 shuttled by PCa‐secreted exosomes, miR‐205‐5p, RUNX2, and SFPQ/PTBP2 as well as their interaction in the osteogenic differentiation of hBMSCs could not be discussed clearly and requires further investigation in future clinical trials.

## CONFLICT OF INTEREST

The authors declare that they have no competing interests.

## ETHICS APPROVAL AND CONSENT TO PARTICIPATE

Ethics Committee of the First Affiliated Hospital, Sun Yat‐Sen University approved the animal experiments.

## AUTHOR CONTRIBUTIONS

Xiaopeng Mao, Chengqiang Mo and Bin Huang designed the study. Chengqiang Mo, Bin Huang, and Jintao Zhuang contributed equally to this work. Jintao Zhuang and Shengjie Guo were involved in data collection. Shuangjian Jiang and Xiaopeng Mao performed the statistical analysis and preparation of figures. Chengqiang Mo and Xiaopeng Mao drafted the paper. All authors read and approved the final manuscript.

## Supporting information


**FIGURE S1** NEAT1 promoted osteogenic differentiation of hBMSCs. (A) The expression of NEAT1 in hBMSCs transfected with NEAT1 overexpression plasmid was detected by RT‐qPCR. (B) CCK‐8 kit was used to detect the growth ability of MSC after 7 days of culture, NS: not significant. (C) ALP activity was measured in hBMSCs on the seventh day in proliferation medium (PM) and OM media. (D) ARS mineralization was measured in MSC on the 14th day in PM and OM media. (E) RT‐qPCR and western blot were used to detect the relative mRNA and protein expression of RUNX2 and ALP in PM and OM on the seventh day. (F) RT‐qPCR and western blot were employed to detect the relative mRNA and protein expression of COL1A1 and OCN in PM and OM on the 14th day. ALP, alkaline phosphosphatidium. ARS, alizarin red S; PM, proliferative medium; OM, osteogenic medium; RUNX2, runt related transcription factor 2; COL1A1, collagen type I alpha 1 chain; OCN, osteocalcin. *p < 0.05 versus the NC (overexpression negative control) group. Measurement data were expressed as mean ± standard deviation, and unpaired *t*‐test was used for two independent samples. Two‐way ANOVA was used for comparing data at different time points followed by Tukey's post hoc test.

**FIGURE S2** NEAT1 shuttled by MDA‐PCa‐2b‐derived exosomes affected osteogenic differentiation of hBMSCs by regulating RUNX2. hBMSCs were treated with RUNX2 knockdown and MDA‐PCa‐2b‐derived exosomes with NEAT1 overexpression. (A) Expression of NEAT1 and miR‐205‐5p and RUNX2 protein level were assessed by RT‐qPCR and western blot analysis. (B) ALP activity of hBMSCs on the seventh day was detected by ALP staining. (C) ARS mineralization assay was performed on hBMSCs on the 14th day. (D) RT‐qPCR and western blot were used to detect the relative mRNA and protein expression of RUNX2 and ALP on the seventh day. (E) RT‐qPCR and western blot were used to detect the relative mRNA and protein expression of COL1A1 and OCN on the 14th day. *p < 0.05 versus MDA‐PCa‐2b‐NC + sh‐NC group. #*p* < 0.05 versus MDA‐PCa‐2b‐NEAT1 + sh‐NC group. Data are shown as the mean ± standard deviation of three technical replicates. Data among multiple groups were compared using one‐way ANOVA, and pairwise comparison was conducted using Tukey.

**FIGURE S3** Rab27a expression was downregulated in MDA‐PCa‐2b cells. Rab27a expression was downregulated in MDA‐PCa‐2b cells treated with sh‐Rab27a determined by RT‐qPCR and western blot analysis. *p < 0.05 versus sh‐NC group. Data are shown as the mean ± standard deviation of three technical replicates. Unpaired *t*‐test was used for analysis of differences between two groups.

**FIGURE S4** Schematic diagram of tibial injection of PCa cells. hBMSCs and C4‐2B cells were mixed at a ratio of 1:1 for tibial injection.

Supporting Information
