# Supplementary figures and images for: LncRNA nuclear‐enriched abundant transcript 1 shuttled by prostate cancer cells‐secreted exosomes initiates osteoblastic phenotypes in the bone metastatic microenvironment via miR‐205‐5p/runt‐related transcription factor 2/splicing factor proline‐ and glutamine‐rich/polypyrimidine tract‐binding protein 2 axis
Source: Clin Transl Med. 2021 Aug 9;11(8):e493. doi: 10.1002/ctm2.493 (PMC8351523; doi:10.1002/ctm2.493)

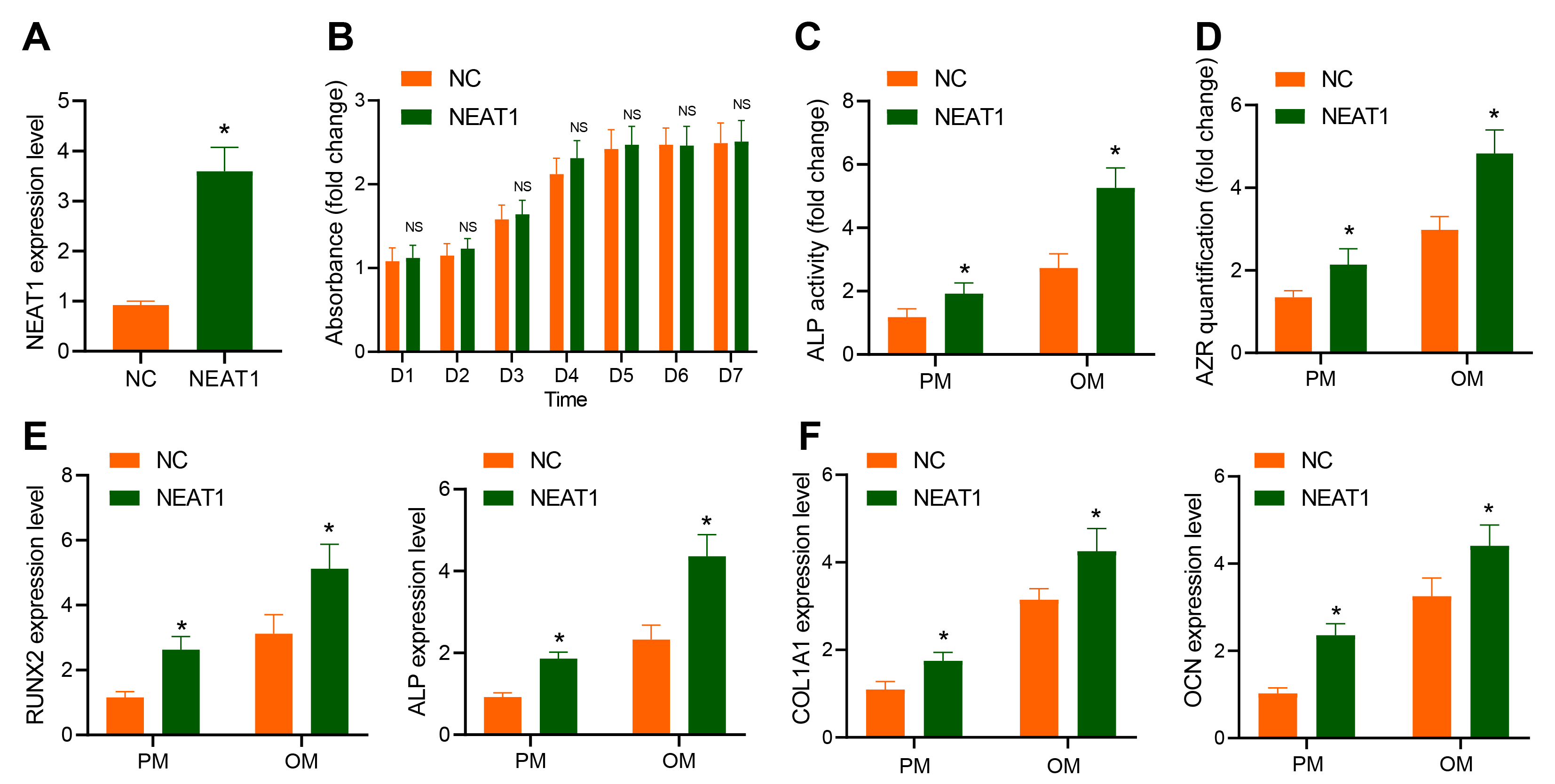

Supplement: Supplementary file 1 — FIGURE S1 NEAT1 promoted osteogenic differentiation of hBMSCs. (A) The expression of NEAT1 in hBMSCs transfected with NEAT1 overexpression plasmid was detected by RT‐qPCR. (B) CCK‐8 kit was used to detect the growth ability of MSC after 7 days of culture, NS: not significant. (C) ALP activity was measured in hBMSCs on the seventh day in proliferation medium (PM) and OM media. (D) ARS mineralization was measured in MSC on the 14th day in PM and OM media. (E) RT‐qPCR and western blot were used to detect the relative mRNA and protein expression of RUNX2 and ALP in PM and OM on the seventh day. (F) RT‐qPCR and western blot were employed to detect the relative mRNA and protein expression of COL1A1 and OCN in PM and OM on the 14th day. ALP, alkaline phosphosphatidium. ARS, alizarin red S; PM, proliferative medium; OM, osteogenic medium; RUNX2, runt related transcription factor 2; COL1A1, collagen type I alpha 1 chain; OCN, osteocalcin. *p < 0.05 versus the NC (overexpression negative control) group. Measurement data were expressed as mean ± standard deviation, and unpaired t‐test was used for two independent samples. Two‐way ANOVA was used for comparing data at different time points followed by Tukey's post hoc test. [file CTM2-11-e493-s005.jpg]

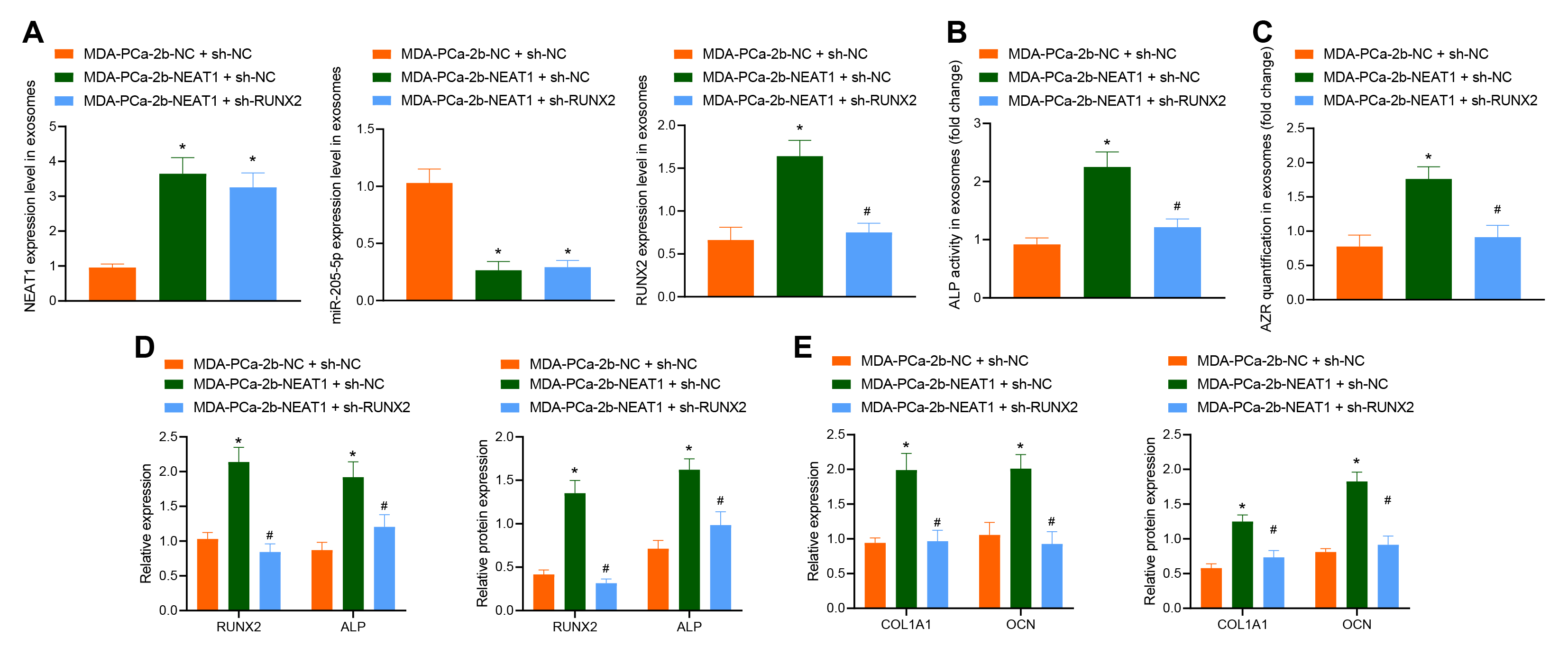

Supplement: Supplementary file 2 — FIGURE S2 NEAT1 shuttled by MDA‐PCa‐2b‐derived exosomes affected osteogenic differentiation of hBMSCs by regulating RUNX2. hBMSCs were treated with RUNX2 knockdown and MDA‐PCa‐2b‐derived exosomes with NEAT1 overexpression. (A) Expression of NEAT1 and miR‐205‐5p and RUNX2 protein level were assessed by RT‐qPCR and western blot analysis. (B) ALP activity of hBMSCs on the seventh day was detected by ALP staining. (C) ARS mineralization assay was performed on hBMSCs on the 14th day. (D) RT‐qPCR and western blot were used to detect the relative mRNA and protein expression of RUNX2 and ALP on the seventh day. (E) RT‐qPCR and western blot were used to detect the relative mRNA and protein expression of COL1A1 and OCN on the 14th day. *p < 0.05 versus MDA‐PCa‐2b‐NC + sh‐NC group. #p < 0.05 versus MDA‐PCa‐2b‐NEAT1 + sh‐NC group. Data are shown as the mean ± standard deviation of three technical replicates. Data among multiple groups were compared using one‐way ANOVA, and pairwise comparison was conducted using Tukey. [file CTM2-11-e493-s002.jpg]

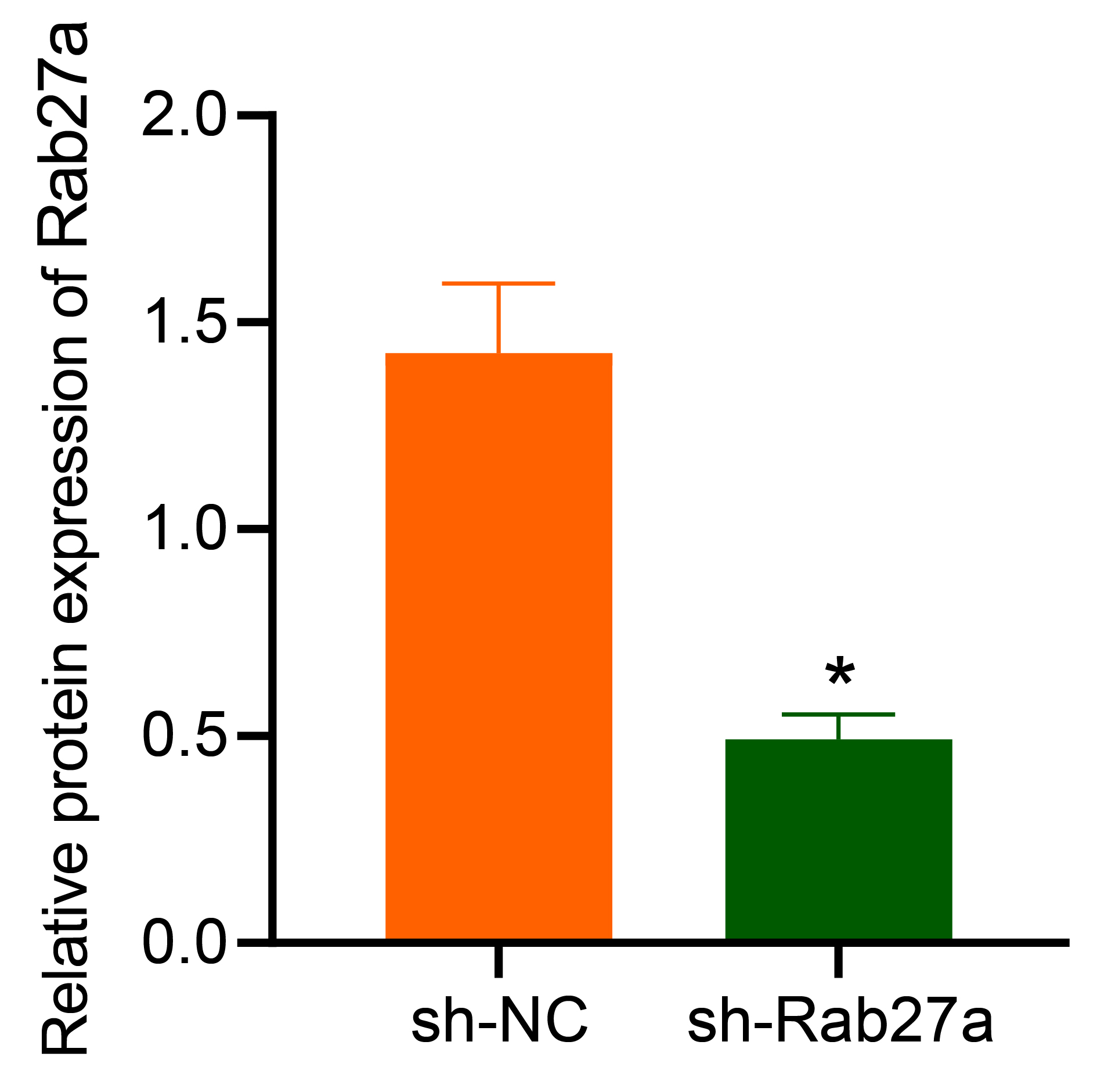

Supplement: Supplementary file 3 — FIGURE S3 Rab27a expression was downregulated in MDA‐PCa‐2b cells. Rab27a expression was downregulated in MDA‐PCa‐2b cells treated with sh‐Rab27a determined by RT‐qPCR and western blot analysis. *p < 0.05 versus sh‐NC group. Data are shown as the mean ± standard deviation of three technical replicates. Unpaired t‐test was used for analysis of differences between two groups. [file CTM2-11-e493-s003.jpg]

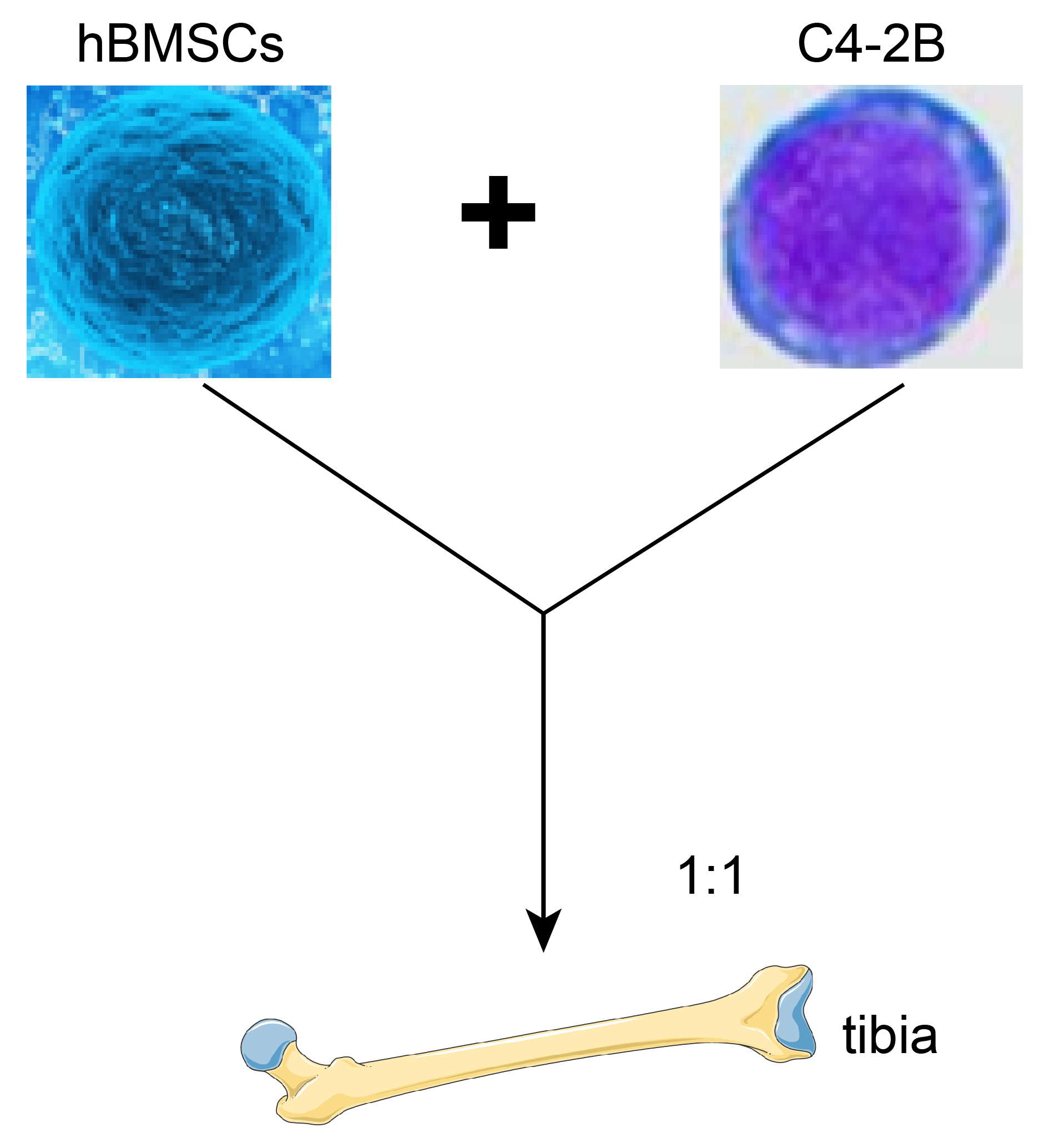

Supplement: Supplementary file 4 — FIGURE S4 Schematic diagram of tibial injection of PCa cells. hBMSCs and C4‐2B cells were mixed at a ratio of 1:1 for tibial injection. [file CTM2-11-e493-s001.jpg]
