# Supplementary material for: LncRNA nuclear‐enriched abundant transcript 1 shuttled by prostate cancer cells‐secreted exosomes initiates osteoblastic phenotypes in the bone metastatic microenvironment via miR‐205‐5p/runt‐related transcription factor 2/splicing factor proline‐ and glutamine‐rich/polypyrimidine tract‐binding protein 2 axis
Source: Clin Transl Med. 2021 Aug 9;11(8):e493. doi: 10.1002/ctm2.493 (PMC8351523; doi:10.1002/ctm2.493)
Supplement: Supplementary file 5 — Supporting Information [file CTM2-11-e493-s004.docx]

**Supplementary Table 1** RT-qPCR primer sequences

| Gene | Primer sequence (5’-3’) | |
| --- | --- | --- |
| NEAT1 | F: GCTGGACCTTTCATGTAACGGG | R: TGAACTCTGCCGGTACAGGGAA |
| miR-205-5p | TaqMan Advanced miRNA Assays |  |
| RUNX2 | F: CCCAGTATGAGAGTAGGTGTCC | R: GGGTAAGACTGGTCATAGGACC |
| Rab27a | F: AAGGGATAGAGCACAGCGAG | R: TGCAGTGTAGCGTCCTTAGC |
| U6 | TaqMan RNU6B miRNA quantitative PCR kit (MTT01501) |  |
| GAPDH | F: GTCTCCTCTGACTTCAACAGCG | R: ACCACCCTGTTGCTGTAGCCAA |
| ALP | F: GCACCGCCACCGCCTACC | R: CCACAGATTTCCCAGCGTCCTTG |
| COL1A1 | F: GATTCCCTGGACCTAAAGGTGC | R: AGCCTCTCCATCTTTGCCAGCA |
| OCN | F: CCGCAGCTCCCAACCACAAT | R: GCCAGCCTCCAGCACTGTTTA |
| cel-miR-39 | TaqMan cel-miR-39 miRNA quantitative PCR kit (MTT01513) |  |

**Note**: F, forward; R, reverse.

**Supplementary Table 2**

| NAME | GS<br> follow link to MSigDB | SIZE | ES | NES | NOM p-val | FDR q-val | FWER p-val | RANK AT MAX | LEADING EDGE |  |  |
| --- | --- | --- | --- | --- | --- | --- | --- | --- | --- | --- | --- |
| GO_PROTEIN_LOCALIZATION_TO_CENTROSOME | GO_PROTEIN_LOCALIZATION_TO_CENTROSOME | 17 | 0.576722 | 1.91788 | 0 | 0.661607 | 0.511 | 685 | tags=29%, list=3%, signal=30% | | |
| GO_NUCLEOTIDE_PHOSPHORYLATION | GO_NUCLEOTIDE_PHOSPHORYLATION | 54 | 0.577712 | 1.712769 | 0.005917 | 1 | 0.977 | 2781 | tags=43%, list=14%, signal=49% | | |
| GO_ADP_METABOLIC_PROCESS | GO_ADP_METABOLIC_PROCESS | 43 | 0.617711 | 1.795403 | 0.006048 | 1 | 0.888 | 2781 | tags=47%, list=14%, signal=54% | | |
| GO_RIBONUCLEOSIDE_DIPHOSPHATE_METABOLIC_PROCESS | GO_RIBONUCLEOSIDE_DIPHOSPHATE_METABOLIC_PROCESS | 60 | 0.515978 | 1.749166 | 0.011928 | 1 | 0.957 | 2781 | tags=37%, list=14%, signal=43% | | |
| GO_INTRA_S_DNA_DAMAGE_CHECKPOINT | GO_INTRA_S_DNA_DAMAGE_CHECKPOINT | 12 | 0.566597 | 1.622633 | 0.02008 | 1 | 0.997 | 3004 | tags=50%, list=15%, signal=59% | | |
| GO_ATP_GENERATION_FROM_ADP | GO_ATP_GENERATION_FROM_ADP | 36 | 0.626944 | 1.677359 | 0.024048 | 1 | 0.99 | 2781 | tags=50%, list=14%, signal=58% | | |
| GO_DNA_REPLICATION_CHECKPOINT | GO_DNA_REPLICATION_CHECKPOINT | 14 | 0.649851 | 1.593739 | 0.02459 | 1 | 1 | 3004 | tags=57%, list=15%, signal=67% | | |
| GO_DNA_DEPENDENT_DNA_REPLICATION | GO_DNA_DEPENDENT_DNA_REPLICATION | 89 | 0.538057 | 1.631399 | 0.025692 | 1 | 0.996 | 2542 | tags=42%, list=13%, signal=47% | | |
| GO_NUCLEOSIDE_DIPHOSPHATE_METABOLIC_PROCESS | GO_NUCLEOSIDE_DIPHOSPHATE_METABOLIC_PROCESS | 77 | 0.443957 | 1.606163 | 0.02907 | 1 | 0.999 | 2781 | tags=32%, list=14%, signal=38% | | |
| GO_HIGH_DENSITY_LIPOPROTEIN_PARTICLE_REMODELING | GO_HIGH_DENSITY_LIPOPROTEIN_PARTICLE_REMODELING | 15 | 0.707112 | 1.648096 | 0.032587 | 1 | 0.996 | 1697 | tags=47%, list=9%, signal=51% | | |
| GO_GLUCOSE_CATABOLIC_PROCESS | GO_GLUCOSE_CATABOLIC_PROCESS | 29 | 0.628783 | 1.58068 | 0.037549 | 1 | 1 | 2781 | tags=52%, list=14%, signal=60% | | |
| GO_HISTONE_EXCHANGE | GO_HISTONE_EXCHANGE | 49 | 0.525964 | 1.542076 | 0.039526 | 1 | 1 | 4293 | tags=55%, list=22%, signal=70% | | |
| GO_TETRAHYDROFOLATE_INTERCONVERSION | GO_TETRAHYDROFOLATE_INTERCONVERSION | 11 | 0.662924 | 1.547532 | 0.041916 | 1 | 1 | 4176 | tags=45%, list=21%, signal=58% | | |
| GO_MITOTIC_CHROMOSOME_CONDENSATION | GO_MITOTIC_CHROMOSOME_CONDENSATION | 13 | 0.70718 | 1.562269 | 0.042553 | 1 | 1 | 1473 | tags=62%, list=7%, signal=66% | | |
| GO_DNA_REPLICATION_DEPENDENT_NUCLEOSOME_ORGANIZATION | GO_DNA_REPLICATION_DEPENDENT_NUCLEOSOME_ORGANIZATION | 31 | 0.623775 | 1.556379 | 0.045545 | 1 | 1 | 4612 | tags=68%, list=23%, signal=88% | | |
| GO_REGULATION_OF_CERAMIDE_BIOSYNTHETIC_PROCESS | GO_REGULATION_OF_CERAMIDE_BIOSYNTHETIC_PROCESS | 11 | 0.543615 | 1.515515 | 0.047131 | 1 | 1 | 2984 | tags=36%, list=15%, signal=43% | | |
| GO_KERATINIZATION | GO_KERATINIZATION | 44 | 0.482027 | 1.545338 | 0.048193 | 1 | 1 | 2214 | tags=32%, list=11%, signal=36% | | |
| GO_STRAND_DISPLACEMENT | GO_STRAND_DISPLACEMENT | 23 | 0.559819 | 1.544571 | 0.048733 | 1 | 1 | 1966 | tags=35%, list=10%, signal=39% | | |
| GO_G2_DNA_DAMAGE_CHECKPOINT | GO_G2_DNA_DAMAGE_CHECKPOINT | 30 | 0.474889 | 1.510326 | 0.049801 | 1 | 1 | 2317 | tags=40%, list=12%, signal=45% | | |
